# Enteric glial adenosine 2B receptor signaling mediates persistent epithelial barrier dysfunction following acute DSS colitis

**DOI:** 10.1038/s41385-022-00550-7

**Published:** 2022-07-22

**Authors:** Vladimir Grubišić, Vedrana Bali, David E. Fried, Holger K. Eltzschig, Simon C. Robson, Michelle S. Mazei-Robison, Brian D. Gulbransen

**Affiliations:** 1 Department of Physiology and Neuroscience program, Michigan State University, East Lansing, MI 48824 USA; 2 Department of Biomedical Sciences and Center for Biomedical Innovation, New York Institute of Technology College of Osteopathic Medicine, Old Westbury, NY 11568 USA; 3 Department of Large Animal Clinical Sciences, College of Veterinary Medicine, Michigan State University, East Lansing, MI 48824 USA; 4 McGovern Medical School, The University of Texas Health Science Center at Houston, Houston, TX 77030 USA; 5 Division of Gastroenterology, Departments of Medicine and Anesthesia, Beth Israel Deaconess Medical Center, Harvard Medical School, Boston, MA 02215 USA

## Abstract

Intestinal epithelial barrier function is compromised in inflammatory bowel disease and barrier dysfunction contributes to disease progression. Extracellular nucleotides/nucleosides generated in gut inflammation may regulate barrier function through actions on diverse cell types. Enteric glia modulate extracellular purinergic signaling and exert pathophysiological effects on mucosal permeability. These glia may regulate inflammation with paracrine responses, theoretically mediated via adenosine 2B receptor (A_2B_R) signaling. As the cell-specific roles of A_2B_Rs in models of colitis and barrier dysfunction are unclear, we studied glial A_2B_Rs in acute dextran sodium sulfate (DSS) colitis. We performed and validated conditional ablation of glial A_2B_Rs in *Sox10*^CreERT2+/−^;*Adora2b*^f/f^ mice. Overt intestinal disease activity indices in DSS-colitis were comparable between *Sox10*^CreERT2+/–^;*Adora2b*^f/f^ mice and littermate controls. However, ablating glial A_2B_Rs protected against barrier dysfunction following acute DSS-colitis. These benefits were associated with the normalization of tight junction protein expression and localization including claudin-1, claudin-8, and occludin. Glial A_2B_R signaling increased levels of proinflammatory mediators in the colon and cell-intrinsic regulation of genes including *Csf3*, *Cxcl1*, *Cxcl10*, and *Il6*. Our studies show that glial A_2B_R signaling exacerbates immune responses during DSS-colitis and that this adenosinergic cell-specific mechanism contributes to persistent gut epithelial barrier dysfunction.

## Introduction

Increased permeability of the gut barrier, colloquially termed “leaky gut”, contributes to the pathophysiology of several gastrointestinal (GI) and extraintestinal disorders including metabolic, neurodegenerative, neurodevelopmental, and psychiatric diseases^1^. Gut barrier dysfunction may play a causative role in inflammatory bowel disease (IBD)^2^, where the debilitating disease is associated with relapsing bouts of acute intestinal inflammation and clinical remission. Persistent barrier dysfunction in apparent remission is associated with ongoing diarrhea and abdominal pain^3^ as well as promoting relapse into active intestinal inflammation^4,5^. Current IBD therapies focus on attaining remission through potent anti-inflammatory and immunomodulating drugs and do not address persistent changes in barrier function. Therapies that improve barrier function might enhance the quality of life and length of remission; however, developing such therapies will require a better understanding of mechanisms that regulate barrier dysfunction following inflammation.

Purinergic signaling regulates inflammatory responses in the intestinal mucosa through time-, cell-, ecto-enzyme-, and receptor-dependent kinetic processes. Active inflammation involves an early surge of the pro-inflammatory danger signal ATP, which is subsequently degraded to adenosine through the actions of ectonucleotidases^6,7^. Enhancing ATP degradation by ectonucleotidases ameliorates tissue damage caused by acute inflammation in animal models of colitis^8^ and deleting genes encoding specific ectonucleotidases of the ENTPD-CD39 family exacerbates colitis in mice^9,10^. These effects may be ascribed, at least in part, to changes in the production of adenosine. Extracellular adenosine accumulates following the hydrolysis of ATP in the acute phases of colitis and is typically considered an endogenous anti-inflammatory molecule that mediates the resolution of inflammation^6,7^. Adenosine exerts its effects through A_1_, A_2A_, A_2B_, and A_3_ receptors in the gut wall^11^. Of these, adenosine 2B receptors (A_2B_Rs) are particularly suited to mediate the effects of high adenosine concentrations in inflammation due to their unique low-affinity properties^12^. Global deletion or treatment with A2B receptor antagonists during experimental colitis is associated with increased weight loss and histologic tissue injury^13^. Similarly, investigation in mice with deletion of A_2B_ receptors on intestinal epithelia is associated with more severe intestinal injury^14^ and treatment with an A_2B_ agonist (BAY 606583) provides protection^15^. However, *Adora2B* gene deletion also reduces the severity of two models of experimental colitis in mice^16^ suggesting pro-inflammatory effects of A_2B_R signaling. The conflicting results may depend on the type of model, the exact regimen of treatment, the composition of the bacterial flora^17^, or heterogeneous cell type-specific A_2B_R signaling. Therefore, understanding the cell-specific roles of A_2B_R signaling is important to understand how adenosine regulates barrier function following inflammation.

Multiple cell types including epithelial cells, resident immune cells, neurons and glia in the enteric nervous system (ENS), and neurons projecting to the gut from the central nervous system coordinate barrier function in the intestine^18^. We recently showed that enteric glia modulate secretomotor functions in the healthy gut epithelium^19^ and contribute to post-inflammatory gut barrier dysfunction by altering the composition of extracellular purines^20^. Enteric glia express A_2B_Rs^11,21,22^, but whether and how glial A_2B_R signaling might contribute to barrier function in health and disease is not understood.

We hypothesized that glial A_2B_R signaling regulates acute intestinal inflammation and thereby impacts persistent gut barrier dysfunction. This concept was tested by creating an enteric glial-specific *Adora2B* ablation mouse model and studying the impact on inflammation and barrier dysfunction induced by dextran sulfate sodium (DSS) colitis. In vivo and in vitro assays were conducted to assess gut permeability, immune profiles, visceromotor sensitivity, behavior, and glial signaling. These data show that glial A_2B_Rs are causative in mediating persistent gut barrier dysfunction after acute colitis though immunomodulatory effects that impact the expression of the epithelial tight junction proteins. These glial mechanisms highlight important cell type-specific actions of A_2B_R signaling that contribute, at least in part, to persistent barrier dysfunction after inflammatory insults.

## Results

### Generation of glial *Adora2b* null mice and characterization of glial A_2B_R signal transduction pathways

**Fig. 1:**
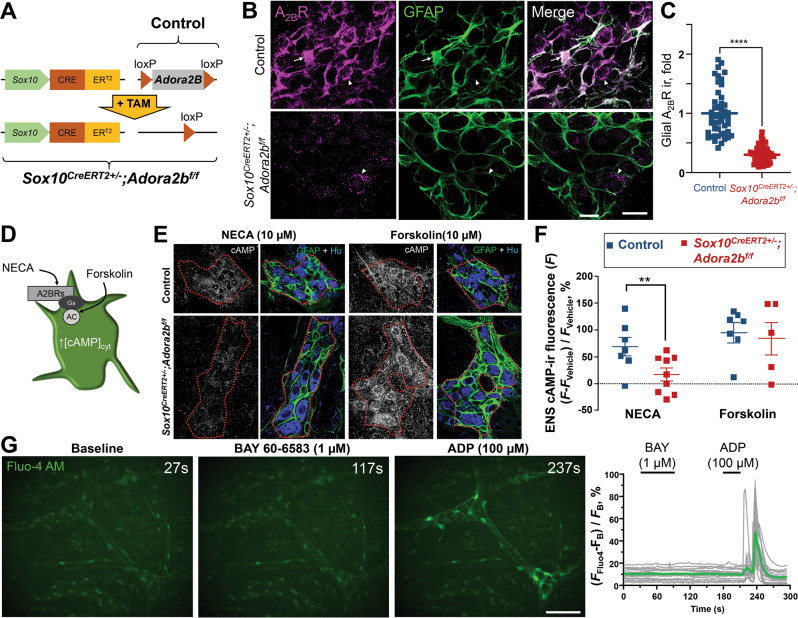
Characterization of glial A_2B_R null mice and glial A_2B_R signal transduction pathways. **A** We generated a novel animal model by crossing the *Sox10*^*CreERT2*^ mouse line with a floxed *Adora2B* gene encoding A_2B_Rs. Tamoxifen treatment (+TAM) ablates *Adora2B* expression from *Sox10* expressing cells. **B** Mouse wholemount myenteric plexus form *Sox10*^*CreERT2+/−*^; *Adora2B*^*f/f*^ mice (bottom) and their control littermates (top) stained with anti-A_2B_R antibody and glial marker anti-GFAP. Arrows point to immunoreactivity (ir) overlay while arrows point to A2BR-ir, expressed in enteric neurons. Scale bar, 20 µm. Note the glial A_2B_R ir depletion of 70 ± 6% (mean ± SEM) in *Sox10*^*CreERT2+/−*^; *Adora2B*^*f/f*^ mice in comparison to their controls (**C**). **C** Glial A_2B_R ir analysis. ****P < 0.0001, unpaired *t* test with Welch’s correction. *N* = 48 and 69 glial cells per group. **D**–**F** Production of cAMP in the mouse myenteric plexus. **D** A schematic showing A_2B_R coupling to Gs protein and production of cAMP with sites of action of a high-affinity adenosine receptor agonist NECA and an adenylyl cyclase (AC) activator Forskolin. **E** Wholemount preparation from *Sox10*^*CreERT2+/−*^; *Adora2B*^*f/f*^ mice (bottom) and their controls (top) were incubated with 10 µM NECA (left), 10 µM Forskolin (right), or vehicle (not shown), and stained with anti-cAMP (gray) antibody and glia/neuron markers anti-GFAP (green) and anti-HuC/D (blue). Anti-GFAP and anti-HuC/D ir were used to identify borders of the myenteric plexus (red dotted line). Scale bars, 25 µm. **F** Intensity of cAMP ir within the myenteric plexus normalized to vehicle controls. **P = 0.004, 2-way ANOVA, Sidak’s multiple comparisons test. *N* = 5–9 mice per group. **G** Cytosolic calcium dynamics. Whole-mount preparations from WT controls were loaded with Fluo4 calcium indicator dye and timelapse imaging was performed (Movie S1). After the initial acquisition of the baseline fluorescence (left image, 27 s), the preparations were incubated with A2BR highly selective agonist BAY 60-6583 (middle image, 117 s) and then ADP as a positive control (right image, 237 s). The graph shows the change in the background-subtracted Fluo4 fluorescence (*F*_Fluo4_ - *F*_B_) normalized to the background fluorescence (*F*_B_) of individual cells traces (gray) and the average trace (green). Scale bar, 50 µm.

We began by crossing *Sox10*^*CreERT2*^ mice with *Adora2b*^*f/f*^ mice to generate a mouse model where A_2B_Rs are conditionally ablated in enteric glial following exposure to tamoxifen (*Sox10*^*CreERT2+/−*^*; Adora2b*^*f/f*^; Fig. 1A). Immunostaining with antibodies against A_2B_Rs demonstrated robust expression in GFAP+ enteric glia at the level of the myenteric plexus in samples from control animals (Fig. 1B). Glial A_2B_R labeling was absent in preparations from tamoxifen-treated *Sox10*^*CreERT2+/−*^*; Adora2b*^*f/f*^ mice and A_2B_R immunoreactivity in enteric neurons was still observed in these animals (Fig. 1B), indicating that the model was effective at selectively deleting glial, and not neuronal, A_2B_Rs. This approach induced a 70 ± 6 % (mean ± SEM; *P* < 0.0001, Welch’s *t*-test) reduction of A_2B_R immunoreactivity in *Sox10*^*CreERT2+/−*^*; Adora2b*^*f/f*^ glia in comparison to their WT littermates (Fig. 1C).

A_2B_Rs couple to Gs and Gq signal transduction pathways in different cell types^23^. Prior work on cultured glial cells suggested that glial A_2B_Rs are responsible for much of the cAMP production in the myenteric plexus^22^; however, some cell lines express A_2B_Rs coupled with Gq^24–26^. We tested which second messenger system couples with A_2B_Rs in enteric glia by assessing the ability of A_2B_Rs to stimulate cAMP and Ca^2+^. We began by investigating in situ cAMP production in the colonic myenteric plexus of tamoxifen-treated *Sox10*^*CreERT2+/−*^*; Adora2b*^*f/f*^ mice and their littermate controls (Fig. 1D–F). Both groups generated comparable amounts of cAMP when stimulated with the adenylyl cyclase activator forskolin (*P* = 0.183, 2-way ANOVA, Sidak’s multiple comparisons test). However, cAMP responses to the high-affinity adenosine receptor agonist NECA were significantly less in samples from mice lacking glial A_2B_Rs (21 ± 15 and 92 ± 20% fluorescence increase from vehicle controls, mean ± SEM, *P* = 0.004, 2-way ANOVA, Sidak’s multiple comparisons test). Ca^2+^ imaging in live preparations of colon myenteric plexus (Fig. 1G, Movie S1) and primary enteric glia (Fig. S1) showed that NECA and the selective A_2B_R agonist BAY 60–6583 did not elicit glial Ca^2+^ responses, although glia did exhibit robust responses to the P2Y_1_ receptor agonist ADP as described previously^27^. This outcome suggests that A_2B_Rs expressed by mouse myenteric glia couple to G_s_ and not G_q_ proteins. Further, we show that this signal cascade is disrupted in mice lacking glial A_2B_Rs.

### Effects of glial A_2B_R ablation on acute DSS colitis

**Fig. 2:**
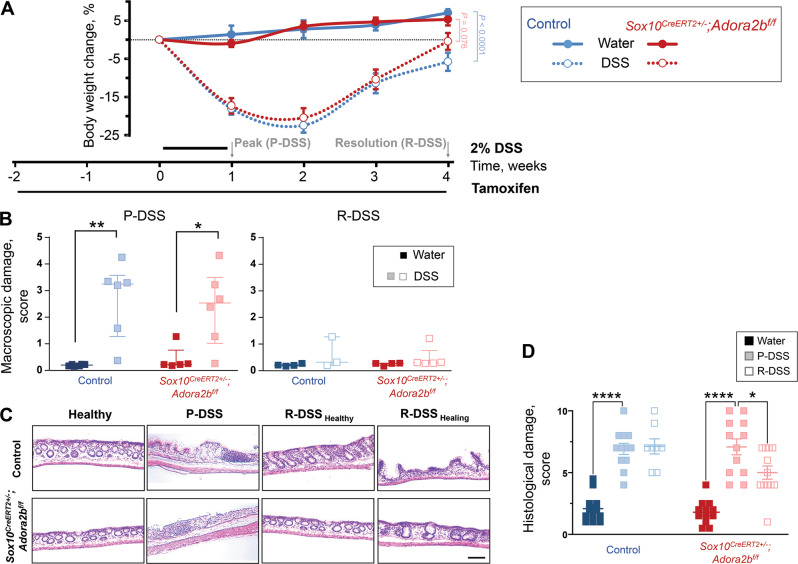
Effects of glial A_2B_R ablation on acute DSS colitis. **A** Body weight change and time course of the experiments. All animals were fed tamoxifen diet for 2 weeks prior and subgroups received 2% DSS in drinking water for a week. DSS-treated animals lose body weight during the DSS treatment and begin weight gain one week after the treatment. Some statistical comparisons are omitted for clarity (see text for details). The body weight changes were used to inform other experiments and identify two-time points reflecting the peak and the resolution of inflammation. **B** Macroscopic damage score at peak (P-DSS, left) and resolution (R-DSS, right) of DSS colitis. *P = 0.014, **P = 0.001, 2-way ANOVA, Sidak’s multiple comparisons test. *N* = 5–6 (left) and 3–5 (right) mice per group. **C** Hematoxylin and Eosin (H&E) stained cross-sections of distal colon from *Sox10*^*CreERT2+/−*^; *Adora2B*^*f/f*^ mice (bottom) and their control littermates (top) that received only regular drinking water (Healthy) or were treated with DSS and assessed at the peak (P-DSS) or resolution (R-DSS) at different healing levels (R-DSS _Healthy_ R-DSS _Healing_). Scale bar = 100 µm. **D** Evaluation of histological staining. See Table S4 for details about histological disease activity scoring. *P = 0.031; ****P < 0.0001; 2-way ANOVA, Tukey’s multiple comparisons test. *N* = 8–12 distal colon sections per group.

*Sox10*^*CreERT2+/−*^*; Adora2b*^*f/f*^ mice and littermate controls were subjected to dextran sodium sulfate (DSS) colitis to test how glial A_2B_R signaling contributes to barrier function following inflammation. DSS disrupts the epithelial barrier^28^ and induces immune gene networks involved in the pathogenesis of IBD^29^. The low dose of DSS (2%) used in these experiments induced comparable weight loss in *Sox10*^*CreERT2+/−*^*; Adora2b*^*f/f*^ and control mice (*P* > 0.125, 2-way ANOVA, Tukey’s multiple comparisons test; Fig. 2A) without causing severe inflammation and mortality, which could confound interpretations.

Both groups exhibited similar initial weight gain with recovery, but only *Sox10*^*CreERT2+/−*^*; Adora2b*^*f/f*^ mice had fully recovered weight at three weeks post-DSS while their controls did not (*P* = 0.076 and *P* < 0.0001, 2-way ANOVA, Tukey’s multiple comparisons test between healthy and DSS-treated groups). Two time points reflecting the active inflammatory phase of peak DSS-colitis (“P-DSS”; 7 days) and the resolution phase (“R-DSS”; 4 weeks) were chosen for further analysis. Mice lacking glial A_2B_Rs exhibited comparable macroscopic damage to controls at time of peak colitis and no differences in macroscopic damage were detected with resolution (*P* < 0.05 and *P* > 0.05, respectively, 2-way ANOVA and Sidak’s multiple comparisons test between healthy and DSS-treated groups; Fig. 2B). Blinded histological scoring of H&E stained mouse distal colon sections showed comparable damage between glial A_2B_R deficient mice and their controls at peak colitis (Fig. 2C, D). Following resolution, mice lacking glial A_2B_Rs showed significant healing while their controls did not (*P* = 0.0309 and *P* = 0.9998, respectively, 2-way ANOVA and Tukey’s multiple comparisons test between peak and resolution of DSS-colitis groups).

Spleen weights were significantly increased at time of peak colitis in control mice and this effect was noted where glial A_2B_Rs were present (Fig. S2A). Therefore, the loss of glial A_2B_Rs prevents the activation of the immune system during acute inflammation.

Fecal output and composition were measured during the resolution phase to assess possible gross, lasting effects on gut motor and secretory function (Fig. S2B, C). Fecal pellet mass was comparable between all groups (*P* > 0.259, 2-way ANOVA, Sidak’s multiple comparisons test, Fig. S2B), but the fecal fluid content was significantly higher in mice that underwent DSS regardless of genotype (*P* < 0.006, 2-way ANOVA, Sidak’s multiple comparisons test, Fig. S2C). This suggests that long-lasting functional changes at the level of the mucosa are still present.

### Glial A_2B_Rs mediate persistent gut barrier dysfunction

**Fig. 3:**
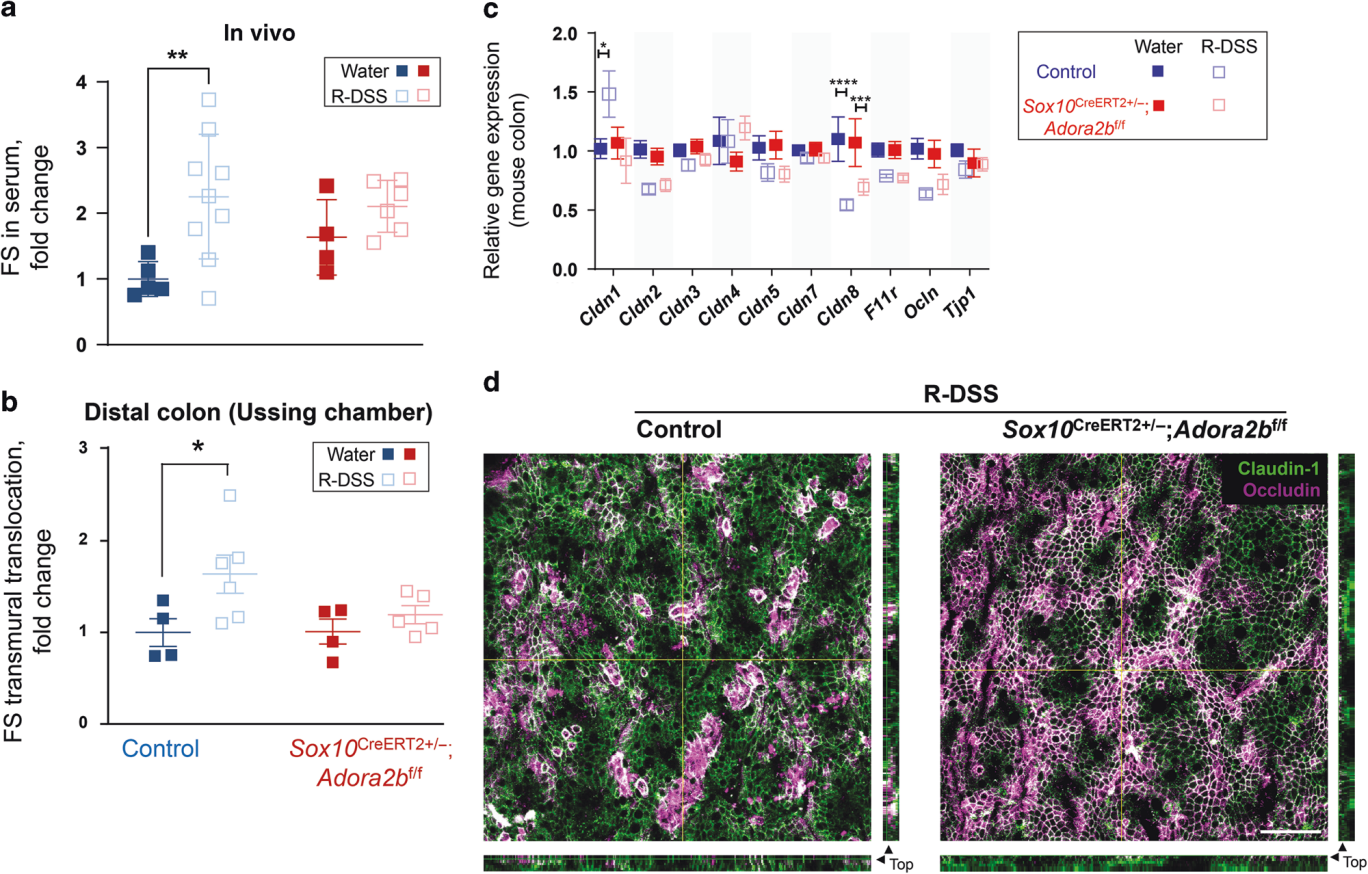
Glial A_2B_Rs mediate persistent gut barrier dysfunction after inflammation. **a**, **b** Gut wall permeability to fluorescein-5-(and-6)-sulfonate (FS) in vivo (**a**) and live distal colon preparations (**b**) at the resolution of DSS-induced colitis (R-DSS). **a** Normalized FS fluorescence intensity in mouse serum 5 h after gavage. **P = 0.007, 2-way ANOVA, Sidak’s multiple comparisons test. *N* = 4–9 mice per group. **b** Normalized rate of transmural FS translocation in the mouse distal colon. *P = 0.032, 2-way ANOVA, Sidak’s multiple comparisons test. *N* = 4–6 mice per group. **c** Transcript expression of the tight junction genes from the mouse colon homogenates. *P = 0.0103; ***P = 0.0003; ****P < 0.0001; 2-way ANOVA, Tukey’s multiple comparisons test. **d** Confocal micrographs of the surface of the Claudin-1 and Occludin stained colon mucosa (green and magenta) at the resolution of DSS-colitis (R-DSS). Occludin expression is relatively evenly distributed in the *Sox10*^*CreERT2+/−*^; *Adora2B*^*f/f*^ mucosa (right) compared with the with the aggregated expression observed in controls (left). Scale bar = 50 µm.

In vivo and ex vivo assays of gut permeability were conducted to specifically assess how glial A_2B_R signaling contributes to persistent barrier dysfunction following colitis (Fig. 3a, b, S3). Following the resolution of colitis, control mice exhibited increased gut permeability as assessed by fluorescein-5-(and-6)-sulfonate flux in vivo and in Ussing chamber experiments (*P* = 0.007 and 0.032, for in vivo and ex vivo, 2-way ANOVA, Sidak’s multiple comparisons test; Fig. 3a, b). Remarkably, mice lacking glial A_2B_Rs were completely protected against residual gut permeability following colitis (*P* = 0.502 and 0.717, for in vivo and ex vivo, 2-way ANOVA, Sidak’s multiple comparisons test). This observation suggests that glial A_2B_R signaling contributes to the development of persistent barrier dysfunction after acute inflammation, which is opposite to the previously observed protective effect of the enterocyte-specific A_2B_R signaling^14^. No sex differences were observed in these experiments, so all other experiments were carried out with mixed sexes. Of note, in vitro transmural conductance recordings following resolution of colitis did not show significant DSS treatment-induced effects (*P* > 0.281, 2-way ANOVA, Sidak’s multiple comparisons test; Fig. S3A) indicating possible compensatory downregulation of ion channels. However, additional in vivo permeability experiments using a 4 kDa FITC-dextran (Fig. S3B) recapitulated findings previously observed with the 0.4 kDa fluorescein-5-(and-6)-sulfonate (Fig. 3a, b).

The main components of the gut epithelial barrier are epithelial cells and tight junctions that seal paracellular pathways between the cells^30^. Given that tissue damage was comparable between animals regardless of genotype, we reasoned that changes in the expression of tight junction proteins might underlie the increased permeability during resolution. Gene expression analysis showed that several tight junction members are altered during the resolution phase of colitis (Fig. 3c). Of these, *Cldn1* and *Cldn8*, encoding claudin-1 and claudin-8, were most impacted and were regulated in a reciprocal manner. *Cldn1* transcription was significantly increased in DSS-treated control mice while *Cldn8* transcripts were decreased (*P* = 0.010 and *P* < 0.0001, 2-way ANOVA, Tukey’s multiple comparisons test). Interestingly, mice lacking glial A_2B_Rs were protected against the upregulation of *Cldn1* and seemed to exhibit less change in *Cldn8* (*P* = 0.912 and 0.0003, 2-way ANOVA, Tukey’s multiple comparisons test). Similarly, the occludin encoding gene *Ocln* showed a trend for a larger transcription decrease in DSS-treated controls than mice with glial A_2B_R deletion (*P* = 0.059 and 0.106, 2-way ANOVA, Tukey’s multiple comparisons test). Since *Cldn1* overexpression can lead to barrier dysfunction by disrupting the trafficking of other proteins^31^, we performed a confocal analysis of claudin-1 and occludin protein localization in whole-mount preparations of mucosa in the 12 µm of mucosa closest to the gut lumen (Fig. 3d). While claudin-1 was evenly distributed in the mucosal surface, DSS-treated controls showed occludin aggregates and areas without occludin expression (Fig. 3d, left) and both proteins were evenly distributed in *Sox10*^*CreERT2+/−*^*; Adora2b*^*f/f*^ mucosal preparations (Fig. 3d, right). Together, these findings show that altered tight junction gene expression and protein localization contribute to increased barrier permeability following colitis and that these changes involve signaling through glial A_2B_Rs.

Increased barrier permeability contributes to the pathophysiology of irritable bowel syndrome which includes abdominal pain and depression^3,32,33^. We investigated whether either effect was present in this animal model by assessing visceromotor responses to colorectal distension (Fig. S4) and behavioral assays that test social interaction as a measure of depressive-like behavior in mice (Fig. S5). Three weeks after DSS, mice exhibited comparable visceromotor responses and social behavior as healthy controls. Hence, albeit 2% DSS does cause visceral hypersensitivity during acute colitis^34^, this effect does not persist for several weeks after inflammation has resolved.

### Ablating glial A_2B_Rs does not affect cell density in the myenteric plexus, cell proliferation, or localization

**Fig. 4:**
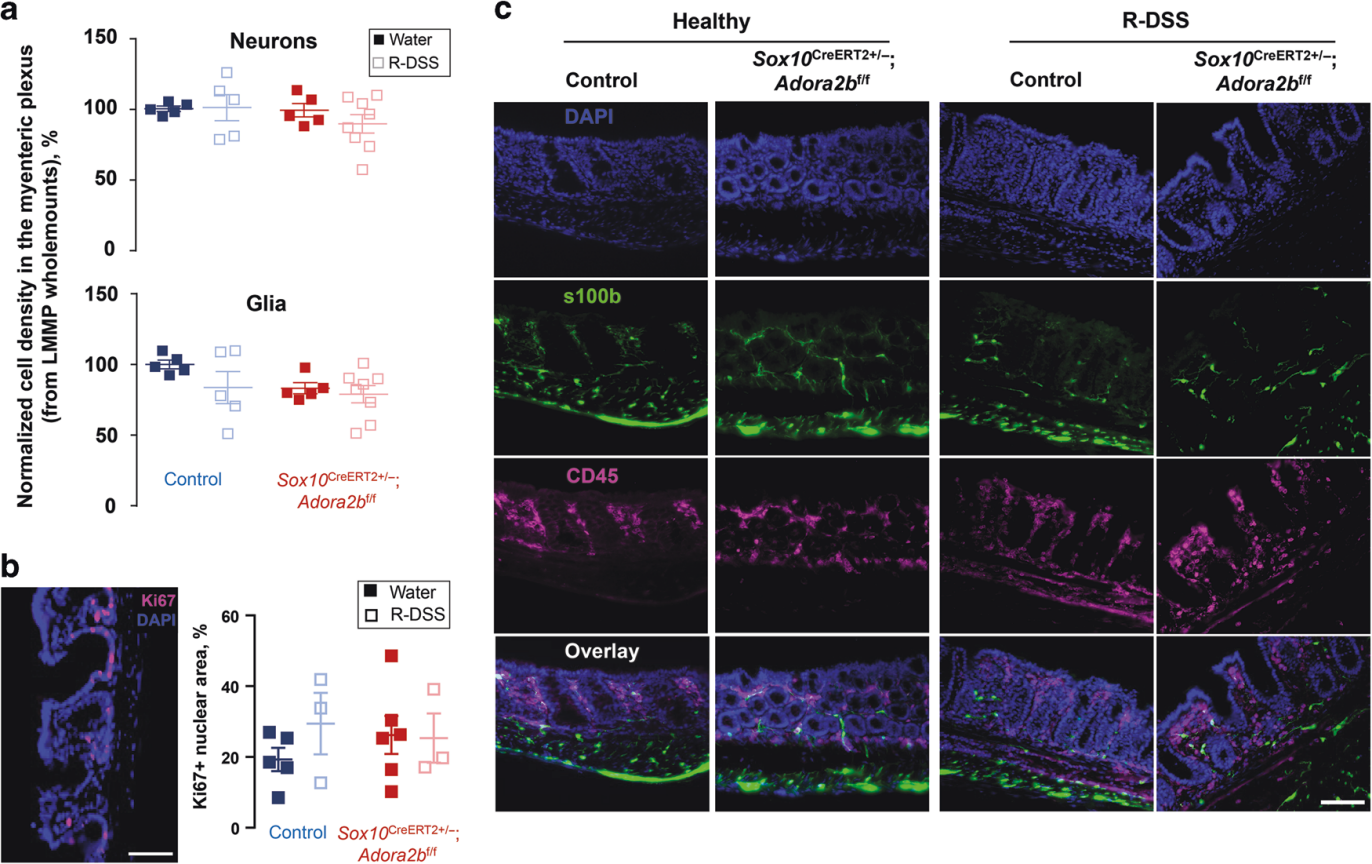
Glial A_2B_Rs do not affect cell density in the myenteric plexus, cell proliferation, or localization during resolution from DSS colitis. **a** Neuron and glia packing density in the myenteric plexus of the mouse colon. Data were obtained from longitudinal muscle myenteric plexus whole-mounts stained with neuronal marker HuC/D and glial marker GFAP (not shown). **b** Cell proliferation was assessed from intestinal cross-sections stained with proliferation marker Ki67 (magenta) and counterstained with DAPI (blue). **c** Intestinal cross-sections were stained with glial marker s100b (green) and immune cell marked CD45 (magenta) and counterstained with DAPI. Notice the proximity of enteric glia to the intestinal epithelium and resident immune cells. Scale bars = 100 µm.

Astrocytic A_2B_R signaling protects cortical neurons against excitotoxicity^35^, so changes in enteric neuron survival may contribute to the observed effects on permeability. However, assessment of the packing density of Hu+ enteric neurons in the myenteric plexus showed no differences between genotypes and treatments (Fig. 4a). It is also possible that changes in glial proliferation and/or migration to the mucosa could contribute to altered permeability because mucosal glia are born in the myenteric plexus and then migrate into mucosa^36^ and A_2B_Rs have known roles in cell proliferation and migration^37^. Despite this, we observed no differences in glial packing density in the myenteric plexus, overall cell proliferation in the gut wall, or the presence of mucosal glia in any genotype or treatment group (Fig. 4a–c). These data suggest that changes to glial survival and migration to the mucosa, and overt changes to neuron density in the myenteric plexus do not play major roles in persistent permeability induced by DSS or the effects of glial A_2B_Rs ablation.

### Glial A_2B_Rs affect immune mediators in the intestine

**Fig. 5:**
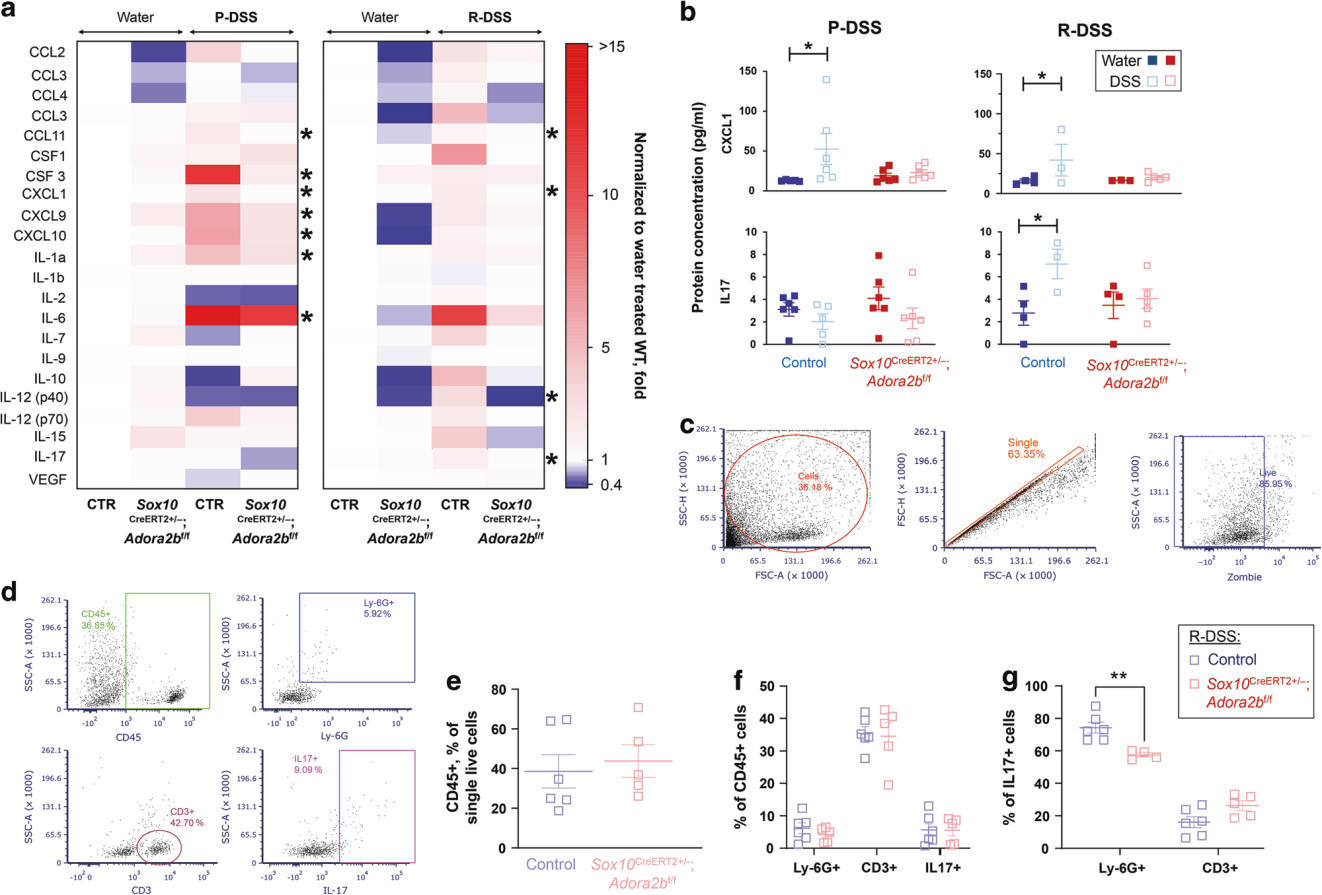
Glial A_2B_Rs affect immune mediators in the intestine. **a**, **b** Multiplex assay of colon homogenates from mice after the peak (P-DSS) and resolution of acute intestinal inflammation (R-DSS). **a** A heatmap showing changes in protein expression relative to healthy controls at P-DSS (left) and R-DSS (right). Shades of blue, white, and red depict decreased, unchanged, and increased protein abundance, respectively. Asterisks mark glial A_2B_R-dependent process as shown in (**b**). **b** Protein concentration of CXCL1 and IL-17 at P-DSS (left) and R-DSS (right). *P < 0.045, one-sided 2-way ANOVA, Sidak’s multiple comparisons test. *N* = 3–6 mice per group. **c**–**g** Flow cytometry of cells dissociated from colon lamina propria from mice at the resolution of the DSS-colitis (R-DSS). **c** Gating strategy for selection of single live cells using forward and side scatter (FSC and SSC). **d** Gating for CD45+, Ly-6G+, CD3+, and IL-17+ cells were adjusted using fluorescence minus one controls. **e**–**g** Summary and analysis of cell populations such as CD45+ (**e**), double positive CD45+ and Ly-6G+, CD3+, or IL-17+ (**f**), and double postitive IL-17+ and Ly-6G+ or CD3+ (**g**). **P = 0.004, 2-way ANOVA, Sidak’s multiple comparisons test. *N* = 4–6 mice per group.

Enteric glia influence immune responses in the intestine^34,38,39^ and are localized with immunocytes near the gut epithelium (Fig. 4c). Based on this and the data showing that ablating glial A_2B_Rs protects against spleen enlargement during the acute phase of DSS colitis (Fig. S2A), we hypothesized that immune cells might serve as an intermediary between glia and effects on gut barrier. To test this hypothesis, we first investigated immune signaling in the gut wall and observed significant effects of glial A_2B_R ablation on cytokine and chemokine expression during both the acute and resolution phases of colitis (Fig. 5a). Ablating glial A_2B_Rs protected against increases in proinflammatory mediators such as CSF3, CCL11, CXCL1, CXCL9, CXCL10, IL-1a, and IL-6 during acute colitis, and protected against changes in CCL11, CXCL1, IL-12(p40), and IL-17 during resolution. Glial A_2B_R signaling was required for both an early and persistent increase in CXCL1 and was required for an increase in IL-17 during resolution (Fig. 5b). To address the possibility that the observed immune changes were caused by effects of the Sox10-driven A_2B_R ablation model on other glial cells of the central (CNS) and peripheral nervous system, we performed an in silico analysis of *Sox10* and *Adora2B* expression in CNS glia. The results of this analysis show that only oligodendrocyte precursor cells could be potentially affected (Fig. S6). We also investigated immune signaling in mouse serum to determine if systemic effects accounted for the changes observed in the colon. Serum cytokine and chemokine signatures (Fig. S7) differed from those observed in the colon and, futermore, had less glial A_2B_R-specific changes than the oserved tissue-specific immunomodulation (Fig. 5a). Together, these data support the conclusion that the observed effects in this model are due to enteric glial-specific changes rather than systemic or CNS-driven effects.

Flow cytometry analysis of the gut lamina propria during resolution showed no changes in numbers of CD45+, CD3+, Ly-6G+, or IL-17+ cells (Fig. 5c–f). Myeloperoxidase levels in colon tissue following resolution were also not significantly different between treatments or genotypes (Fig. S8). However, the proportion of double-positive Ly-6G+ IL-17+ cells was significantly decreased in mice lacking glial A_2B_Rs (Fig. 5g). Taken together, these findings indicate that glial A_2B_Rs increase pro-inflammatory signaling during acute inflammation and stimulate IL-17 production in Ly-6G+ cells. This finding suggests that enteric glia influence neutrophils and that this process required A_2B_R signaling during colitis.

### Glial A_2B_R signaling controls glial production of key immune mediators

**Fig. 6:**
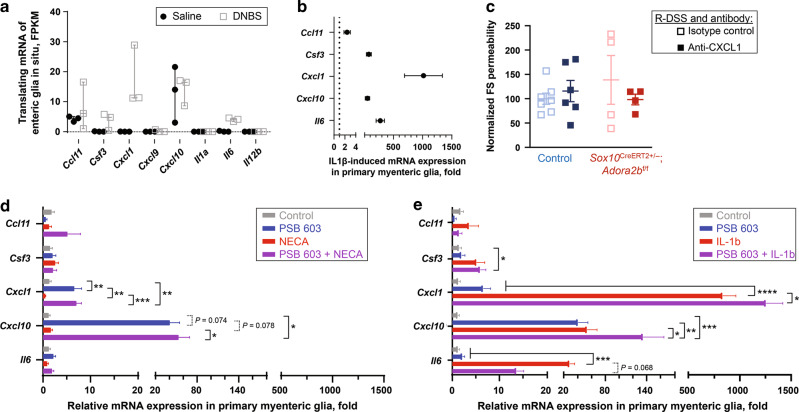
Glial A_2B_R signaling controls the glial production of the key immune mediators. **a** Expression of immune mediators whose whole tissue protein expression was affected by the glial A_2B_R signaling (Fig. 5a) obtained using a previously published dataset of mouse colon in situ glial translating mRNA transcriptome^40^. Data are shown as median ± range. Selected immune mediators that were expressed by enteric glia in saline- and 2,4-dinitrobenzene sulfonic acid (DNBS)-treated mice were further tested on primary glia (**b**). *N* = 3 mice per group. **b** Proinflammatory cytokine IL-1b stimulates the glial production of key immune signaling molecules. Primary myenteric glia were incubated with vehicle or IL-1β. Expression of selected mediators was determined by qPCR and normalized to the vehicle controls. *N* = 6 mice per group. **c** In vivo neutralization with anti-CXCL1 antibody. *N* = 4–7 mice per group. **d**, **e** A_2B_R signaling has diverse and distinct effects on the production of immune mediators in primary enteric glial cells. Cultured cells were stimulated with selective adenosine receptor agonist NECA (**d**) or proinflammatory mediator IL-1β (**e**) alone or in the presence of the selective A2BR inhibitor PSB 603. **d** A2BR signaling suppresses basal expression of *Cxcl1* and *Cxcl10* in cultured enteric glia. *P < 0.013, **P < 0.005; ***P = 0.0009, 2-way ANOVA, Sidak’s multiple comparisons test. *N* = 6–8 mice. **e** IL-1β-induced expression of glial *Il-6* partly relies on A_2B_R signaling while A_2B_R inhibition has additive effects on IL-1β-induced expression of *Csf3*, *Cxcl1*, and *Cxcl10* in primary cultures of enteric glia. *P < 0.046, **P = 0.006, ***P < 0.0004, ****P < 0.0001, 2-way ANOVA, Sidak’s multiple comparisons test. *N* = 6–8 mice. *N* = 6–7 mice per group.

Screening a published RNA sequencing dataset of enteric glial transcriptional responses in health and inflamation^40^ showed that enteric glia express several immune mediators in the colon and that genes for certain mediators are upregulated during inflammation (Fig. 6a). One of these, CXCL1, was also dependent on glial A_2B_R expression (Fig. 5a, b) and contributes to neutrophil recruitment. To test whether glia directly produce the proinflammatory mediators that exhibited A_2B_R dependence in whole colon samples, primary cultures of enteric glia were prepared and the expression of chemokines was measured in basal conditions and after stimulation with pro-inflammatory mediator IL-1β (Fig. 6b). IL-1β-induced potent increases in glial *Ccl11*, *Cxcl10*, *Csf3*, *Il6*, and *Cxcl1* transcription that ranged from 4 to 831-fold changes (ordered by the fold increase).

The large increases in glial *Cxcl1* transcription and tissue CXCL1 production, the dependence on glial A_2B_R signaling, and changes in Ly-6G/IL-17+ cells suggest that glia exert effects on barrier function through actions on neutrophils, which play a key role in DSS colitis^41^ and IBD^42^. Neutralizing CXCL1 protects mice against inflammation in the acute phase of DSS colitis^41^. Given that glial A_2B_R dependent signaling induces a sustained increase in CXCL1 during resolution (Fig. 5b), we tested if neutralizing CXCL1 during the resolution of colitis would protect barrier function (Fig. 6c). A CXCL1 neutralization antibody or its isotype control was injected at the end of the DSS treatment to block the effects of the persistent CXCL1 increase without affecting acute inflammation. No significant differences were observed between treatment groups; however, gut permeability was highly variable in isotype control-treated glial A_2B_R knockout mice that was not observed in anti-CXCL1 treated littermates. The variability in this experiment could suggest that glial A_2B_Rs signaling has a complex role in immune modulation. We tested this in primary glial cultures using the adenosine receptor agonist NECA or IL1-β stimulation alone or together with PSB 603, a highly selective A_2B_R blocker (Fig. 6d, e). The data show that PSB 603 alone increased expression of *Cxcl1* and *Cxcl10* [6.6 ± 1.6 and 40.5 ± 14.2 (mean ± SEM) fold increase from untreated controls, *P* = 0.005 and 0.074, ANOVA, Tukey’s multiple comparisons test] while the selective adenosine receptor agonist NECA had no effect when applied alone [*P* > 0.935 (NECA vs controls), ANOVA, Tukey’s multiple comparisons test] or when combined with A_2B_R inhibition [*P* > 0.846 (PSB 603 vs PSB 603 + NECA), ANOVA, Tukey’s multiple comparisons tests] (Fig. 6d). These outcomes indicate that glial A_2B_R signaling suppresses the basal expression of *Cxcl1* and *Cxcl10*. Major differences were observed in the effects of glial A_2B_R signaling on gene expression for other inflammatory mediators. For instance, IL-1β-induced increases in *Cxcl1* and *Cxcl10* transcription were enhanced by PSB 603 (831.3 ± 129.2 vs 1255 ± 165.7 and 53.6 ± 14.4 vs 134.8 ± 30.1 fold increase from unstimulated controls, *P* = 0.043 and 0.020, ANOVA, Tukey’s multiple comparisons test), but PSB 603 tended to reduce IL-1β induced expression of *Il6* (28 ± 7 vs 14 ± 2 fold increase from unstimulated controls, *P* = 0.068, ANOVA, Tukey’s multiple comparisons test) (Fig. 6e). In addition, *Csf3* expression reached significance only with combined A_2B_R inhibition and IL1b stimulation (5.9 ± 1.2 fold increase from unstimulated controls, *P* = 0.046, ANOVA, Tukey’s multiple comparisons test). These findings indicate that the glial IL-1β-stimulated expression of *Csf3*, *Cxcl1*, *Cxcl10*, and *Il6* is at least partly mediated via A_2B_R signaling. While A_2B_R signaling mediates glial upregulation of *Il6* and downregulation of *Csf3*, *Cxcl1*, and *Cxcl10*, glial A_2B_Rs in colon tissue mediated the DSS colitis-induced increase in protein expression of all these mediators (Table S1). Since immune mediators can be produced by many different cell types such as immunocytes and epithelial/endothelial cells in the gut wall, the net production is an outcome of intercellular communication. IL-6 has pleiotropic effects on various immune and other cells, and CSF3/CXCL1 or CXCL10 are primarily reserved for granulocytes or macrophages/lymphocytes. Taken together, the data indicate that glial A_2B_R signaling mediates broad immunomodulation during basal conditions and acute intestinal inflammation and that the timing of the glial A_2B_R manipulation could have different outcomes on the inflammatory process and post-inflammatory recovery.

## Discussion

**Fig. 7:**
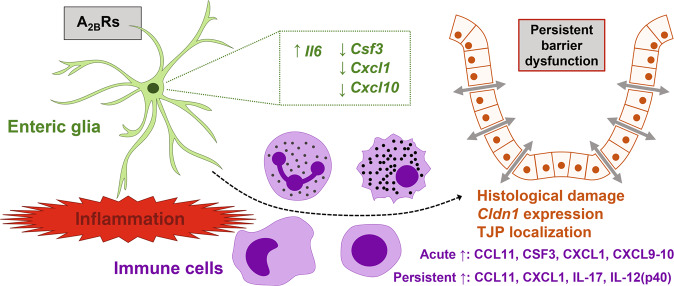
Summary. During acute intestinal inflammation, glial A_2B_R signaling decreases the inflammation-induced expression of *Csf3*, *Cxcl1*, and *Cxcl10*, and increases the expression of *Il6* by enteric glia. Glial A_2B_Rs in turn cause diverse immunomodulation in the inflamed gut leading to persistent epithelial barrier dysfunction through increased histological damage, overexpression of *Cldn1*, and localization of the tight junction proteins (TJP).

The major finding of this study is that enteric glial A_2B_Rs mediate persistent gut barrier dysfunction following acute inflammation through increased histological damage, altered expression of epithelial tight junction proteins, and interactions with the immune system. Proinflammatory signals induce glial expression of several key immune mediators involved in different branches of the immune responses and glial A_2B_R signaling has distinct effects on the inflammation-induced glial expression of immune mediators (Fig. 7).

Prior work assessing the role of enteric glia in gut barrier function provides both evidence for and against significant glial contributions. Initial data from enteric glial ablation models supported a critical role in barrier function since the ablation resulted in fulminant jejunoileitis^43^. This idea was reinforced by in vitro studies which showed that glial mediators that have direct effects on epithelial cells^44,45^. However, no overt “leaky” gut or intestinal inflammation was observed in scenarios where mucosal glia are absent in germ-free mice^36^ or when these cells are ablated using a *Plp1* promoter-driven expression of diphtheria toxin^46^. Combining diphtheria toxin-induced ablation of GFAP+ and Plp1+ cells indicates that these populations of enteric glia have redundant homeostatic roles, but a GFAP+ subpopulation of enteric glia play a particularly important role in the mucosa by promoting epithelial regeneration through effects on the self-renewal of intestinal stem cells^47^. The effects of glia on the epithelial barrier also depend on the extracellular environment and cell signaling mechanism. Activation or suppression of glial Ca^2+^ signaling does not change transepithelial conductance or paracellular permeability in the healthy intestine^19^. However, ablating NTPDase2, an ATP hydrolyze that is predominantly expressed by enteric glia, does increase paracellular permeability during acute DSS colitis^20^. These findings seem to suggest that enteric glia play a much more prominent role in barrier regulation during inflammation. Furthermore, NTPDase2 ablation increased gut permeability following DSS colitis^20^ while deleting glial A_2B_Rs precluded inflammation-induced increase in permeability. These observations indicate that enteric glia play complex roles in purinergic signaling during inflammation and that perturbing components of purine detection or regulation can produce very different functional outcomes.

Purines regulate the immune system by an initial surge of ATP caused by cell death and inflammation, followed by the ecto-enzymatic degradation of ATP which results in the subsequent generation of adenosine^6,7^. Adenosine has a strong effect on tissue damage and repair, and its effects are mediated through the activation of 4 kinds of adenosine receptors: A_1_, A_2A_, A_2B_, and A_3_. While A_1_, A_2A_, and A_3_ are activated by nanomolar concentrations of adenosine, A_2B_R activation requires micromolar levels of adenosine which are mainly present after inflammation^12^. Adenosine A_2B_Rs are usually coupled with G_s_ cascades that produce cAMP, although some cells have A_2B_Rs coupled with G_q_ signaling and downstream intracellular Ca^2+^ signals^24–26^. Experiments using cultured enteric glia suggest that A_2B_Rs are among the main contributors to cAMP production in the myenteric plexus^22^. Our data agree with these observations in culture and show that glial A_2B_Rs stimulate cAMP production in intact myenteric plexus. No calcium responses were observed in response to A_2B_R agonists, supporting the conclusion that glial A_2B_Rs in the mouse myenteric plexus interact with G_s_ protein and contribute to cAMP production.

Deleting A_2B_Rs in enteric glia prevented inflammation-induced intestinal permeability, which indicates a detrimental role for glial A_2B_R signaling that promotes persistent dysfunction following acute DSS colitis. In contrast, ablating A_2B_Rs in gut epithelial cells increased permeability, suggesting that A_2B_Rs in the epithelium provide a protective effect during acute colitis^14^. Adenosine signaling is terminated by uptake from the extracellular space via equilibrative equilibrative nucleoside transporters (ENTs) and deletion of epitheial ENT2 also contributes to gut protection during intestinal inflammation^15^. Taken together, these data suggest that A_2B_R signaling during intestinal inflammation is cell-specific and time-dependent. Ablating glial A_2B_Rs had a striking effect on *Cldn1* expression and the inflammation-induced *Cldn1* upregulation was absent in glial A_2B_R knockout animals. Although claudin-1 is a known barrier-forming protein, its overexpression can prevent proper localization of other tight junction proteins^31^ and could contribute to the observed formation of occludin aggregates which were also glial A_2B_R-dependent. Alternatively, it is possible that the DSS-treated WT mice repaired the damaged epithelium with cells expressing less occludin, making them less sensitive to apoptosis^48^. Understanding how glial A_2B_R signaling affects protein trafficking and subcellular localization of the tight junction proteins would be important for future work in this area.

While enteric glia express mediators that directly affect epithelial regeneration and expression of tight juntions^44,45^, our results suggest that glial A_2B_R signaling affects the intestinal epithelium during inflammation through interactions with the immune system. Gut epithelial tight junctions serve as targets of mucosal immune mediators^30^. For instance, IL-22-induces *Cldn2* upregulation^49^ and TNF-induces occludin endocytosis^50^. Ablating glial A_2B_Rs protected against increases in proinflammatory mediators such as CCL11 (Eotaxin1), CSF3 (G-CSF), CXCL1 (KC), CXCL9 (MIG), CXCL10 (IP10), IL1a, and IL-6 during acute colitis, and protected against changes in CXCL1, CCL11, IL-12(p40), and IL-17 during resolution. CXCL1 was selected for neutralization experiments because IL-1β induced over an 800-fold increase in glial expression of *Cxcl1*, glial expression of *Cxcl1* is mediated by the A_2B_R signaling, and glial A_2B_Rs mediate the persistent increase in IL-17 + neutrophils in the mouse colon lamina propria. IL-17 has effects on intestinal epithelial permability^51^ and in the brain astrocyte-dependent production of CXCL1 mediates neutrophil chemotaxis and the blood-brain-barrier permeability^52^. Indeed, CXCL1 neutralization during peak inflammation prevents neutrophil migration and ameliorates tissue damage^41^, but neutralizing CXCL1 after peak inflammation but did not prevent persistent gut barrier dysfunction. These findings suggest a potential therapeutic window for approaches targeting glia-neutrophil interactions. Indeed, at 3 weeks after DSS treatment glial A_2B_R signaling did not affect the number of neutrophils but rather reduced the IL-17 expression levels (Fig. 5f, g), suggesting that glial A_2B_Rs could contribute to anti-apoptotic signaling that delays neutrophil death. This scenario could be tested by depleting  neutrophils during the reosolution of inflammation. The absence of statistically significant effects in CXCL1 neutralization experiments suggests that effects on gut barrier dysfunction could also be mediated by glial communication with other immune cells. Glial A_2B_R signaling decreased the inflammation-induced expression of *Csf3*, *Cxcl1*, and *Cxcl10*, and increased the expression of *Il6* by enteric glia (Fig. 7). These immune mediators such as *Cxcl10* also contribute to the resolution of inflammation and are critical for intestinal tissue repair^53^. This adds a layer of complexity to the overall effects of glial A_2B_R-mediated immunomodulation. An interesting observation was that isotype control antibody injections concealed the effects of glial A_2B_R ablation on intestinal permeability following DSS colitis. Since the control antibody still binds the Fc receptors (FcR), this outcome suggests that glial A_2B_R signaling-dependent barrier dysfunction following inflammation could be mediated by other FcR-expressing cells including (but not limited to) basophils, B lymphocytes, eosinophils, follicular dendritic cells, macrophages, mast cells, and natural killer cells. Enteric glia are known to interact with B lymphocytes^38^, macrophages^34^, and mast cells^39^, and additional studies are needed to future characterize the role of glia-immune interactions in the recovery of gut functions after acute inflammation.

We showed that glial A_2B_R signaling mediates several important branches of the immune signaling which consequently leads to persistent gut barrier dysfunction. Similarly, every subsequent bout of intestinal inflammation would be less pronounced in the glial A_2B_R knockouts and, thus, their intestinal barrier would be more resilient to chronic inflammation. This hypothesis should be tested in animal models of chronic colitis. Since barrier dysfunction in IBD patients strongly correlates with the probability for relapse into active intestinal inflammation^2^, glial A_2B_Rs could potentially be a specific therapeutic target to prevent  IBD relapses.

In conclusion, glial A_2B_Rs contribute to the development of persistent gut barrier dysfunction by diverse interaction with the immune system which impacts epithelial tight junctions (Fig. 7). These novel cell and molecular mechanisms add insight to events contributing to persistent post-inflammatory barrier dysfunction and could be important for understanding pathophysiological mechanisms that contribute to IBD.

## Materials and methods

### Animals

All experimental protocols were approved by the Institutional Animal Care and Use Committees (IACUC) at Michigan State University (MSU), USA. Mice of both sexes were used at 8–12 weeks of age and were maintained in a temperature-controlled environment (Innovive, San Diego, CA; Innocage system with ALPHA-dri^®^ bedding) on a 12-hour light: dark cycle with access to acidified water and food ad libitum. Transgenic mice were generated on a C57Bl/6 genetic background. *Sox10*^*CreERT2*^ transgenic mice were a gift from Dr. Vassilis Pachnis (The Francis Crick Institute, London, England)^54^ and their effectiveness for targeting enteric glia has been described in prior work^19,40,55^. *Sox10*^*CreERT2*^ mice were bred with *Adora2b*^*f/f*^ mice (Ozgene, Bentley DC, WA, Australia)^14,56,57^, to create a conditional ablation of A_2B_Rs in glia (hereafter referred to as *Sox10*^*CreERT2+/−*^*; Adora2b*^*f/f*^). Littermates not expressing Cre were used as background controls (hereafter referred to as control). Cre recombinase was activated 2 weeks before experiments and induced throughout the study by feeding animals chow containing 400 mg/kg tamoxifen citrate (Envigo, Indianapolis, IN, USA). All mice (including controls) were fed with the tamoxifen diet for the same duration to control for the potential effects of tamoxifen. Genotyping was performed by Transnetyx, Inc (Cordova, TN).

### Dextran Sodium Sulfate (DSS) induced colitis

Acute colitis was induced by adding 2% DSS (colitis grade, M.W. 36–50 kDa; MP Biomedical, Solon, OH) to drinking water (2% w/v) for 7 days. Mice were euthanized at 1 day and 3 weeks after the DSS treatment to capture effects at the peak (P-DSS) and resolution (R-DSS) of inflammation time-points, respectively. Mice were weighed every other day and macroscopic damage of intestinal tissue was assessed as described previously^34^. Spleen weights were also obtained and normalized to body weights.

### Pellet weights and fluid content

Pellet composition was assessed as previously described^27^. Animals were individually housed for 1 h in empty cages and their fecal pellets were collected and weighed (wet weight). Pellets were then dried for 24 h at 60 °C and weighed again to obtain their dry weight. Fluid content was calculated as follows: Fluid content (%) = 100 × (wet weight − dry weight) / wet weight.

### Slide and whole-mount immunohistochemistry (IHC)

Mouse distal colons were fixed overnight in Zamboni’s fixative at 4 °C or for 2 h in 4% paraformaldehyde at room temperature. After rinsing in PBS, the fixed colons were micro dissected to obtain whole-mount preparations of myenteric plexus or cryoprotected in 30% sucrose dissolved in phosphate buffer (m/v) before preparing 20 µm thick frozen sections in the Investigative Histopathology Laboratory at Michigan State University. Immunolabeling was conducted as described below and antibody details are supplied in Tables S2–3. Briefly, slides and whole mounts were rinsed three times (10 min each) in phosphate-buffered saline (PBS) or PBS containing 0.1% Triton X-100 (PBST) followed by a 30 or 60 min incubation in blocking solution [containing 4% normal goat serum (or 5% normal horse serum), 0.1 or 0.4% Triton X-100 and 1% bovine serum albumin]. Primary antibodies were applied overnight at room temperature (RT) or for 48 h at 4 °C before being rinsed three times with PBS. Secondary antibodies were applied for 1 or 2 h at RT. Whole mounts were rinsed with 0.1 M phosphate buffer and mounted on slides with bicarbonate-buffered glycerol (consisting of a 1:3 mixture of 142.8 mM sodium bicarbonate and 56.6 mM carbonate to glycerol).

### Image acquisition and analysis

Epifluorescence images were acquired through the ×20 [(PlanApo, 0.75 numerical aperture (n.a.)] and ×40 (PlanFluor, 0.75 n.a.) objectives of an upright epifluorescence microscope (Nikon Eclipse Ni, Melville, NY) with a Retiga 2000R camera (QImaging, Surrey, BC, Canada) controlled by QCapture Pro 7.0 (QImaging). Confocal images were acquired through the ×40 (UPLFL, 1.3 n.a.) and ×60 (Plan-Apochromat, 1.42 n.a.) oil immersion objectives of an inverted Olympus Fluoview FV1000 microscope (Olympus, Center Valley, PA).

Epifluorescence images were analyzed offline using ImageJ software (National Institutes of Health, Bethesda, MD). Cell counts were performed using the cell counter plug-in of ImageJ software. Enteric neuron and glial cell numbers are presented as ganglionic packing density, calculated by counting the number of HuC/D-immunoreactive neurons or s100β-immunoreactive glia within the defined ganglionic area cell counts, and ganglionic expression data analysis were performed on a minimum of 10 ganglia per animal and averaged to obtain a value for that animal. N values represent the number of animals in each experiment and data is expressed as percent water control or mean intensity units (i.u.). Confocal images were processed by MetaMorph 7.0 software (Molecular Devices, LLC, San Jose, CA, United States) or by FV10-ASW 04.02.02.09 software (Olympus Corporation, Shinjuku, Japan).

### In situ production of cAMP

Live whole mounts of myenteric plexus were micro dissected from the distal colon on ice-cold DMEM/Nutrient Mixture F-12 (Life Technologies) supplemented with L-glutamine and 4-(2-hydroxyethyl)-1-piperazineethanesulfonic acid (HEPES). Then the preparations were placed in an incubator (37 °C in a 95% O_2_ / 5% CO_2_) for 30 min and subsets of preparations received NECA (10 µM), forskolin (10 µM), or vehicle. After a 15 min incubation, samples were fixed with 4% paraformaldehyde (2 h, RT) and stained for cAMP, GFAP, and HuC/D, imaged and analyzed as described above.

### Ca^2+^ imaging

Live samples of colonic myenteric plexus were prepared for Ca^2+^ imaging as previously described^27^. Briefly, colonic segments were collected in ice-cold Dulbecco’s modified Eagle medium (DMEM) and transferred to Sylgard-coated open diamond-shaped bath recording chambers. Tissue segments were opened along the mesenteric border and the mucosa, submucosa, and longitudinal muscle were removed by microdissection to expose the myenteric plexus. The resulting circular muscle myenteric plexus (CMMP) preparations were incubated for 15 min at room temperature in an enzyme mixture consisting of 150 U/mL Collagenase type II and 1 U/mL Dispase (Life Technologies) dissolved in DMEM. CMMP were washed 3 times with DMEM and then loaded with 4 μM Fluo-4 AM, 0.02% Pluronic F-127 and 200 μM water-soluble probenecid (Life Technologies) in DMEM for 45 min at 37°C (5% CO2, 95% air). Whole mounts were superfused with Krebs buffer (37 °C) at 2–3 mL min^−1^ and drugs were bath applied: BAY 60–6583 (10 nM, 1 µM, and 10 µM) or NECA (10 µM), and then ADP (100 µM) as a positive control. Images were acquired every 1 to 2 s through the ×40 water-immersion objective (LUMPlanFI, 0.8 numerical aperture) of an upright Olympus BX51WI fixed stage microscope (Olympus, Center Valley, PA) using IQ2 software or NIS-Elements software (version 4.5) and a Neo sCMOS or an Andor Zyla sCMOS camera (Andor, South Windsor, CT).

### Chemicals and reagents

BAY 60–6583, NECA, and PSB 603 were obtained from Tocris (Bio-Techne Corporation, Minneapolis, MN). Other chemicals and reagents, unless otherwise stated, were purchased from Sigma-Aldrich (St. Louis, MO).

### Histology

Sections of the distal colon were fixed overnight in Zamboni’s fixative at 4 °C. The tissue was washed with PBS until cleared. Hematoxylin and Eosin (H&E) staining was performed on 20 µm thick cross-sections of the distal colon by the Investigative Histopathology Laboratory at MSU. A disease activity assessment was performed as previously published^34^ and criteria for disease activity scoring are identified in Table S4.

### Cytokine/chemokine multiplex array

Mouse colon tissue samples were homogenized on ice using a tissue grinder in a buffer (pH 7.5) containing: Tris/Tris-HCl (25 mM), NaCl (130 mM), KCl (2.7 mM), Tween 20 (5% v/v), and a tablet with protease inhibitor cocktail (Sigma S8820). The samples were sonicated with 10 pulses of 1 s intervals and centrifuged at 10,000 × *g* for 10 min at 4 °C. Supernatant protein concentrations were measured on a NanoDrop™ Lite Spectrophotometer (ThermoFisher Scientific) and samples were adjusted to have the same concentration. An aliquot of each sample was further diluted (2–4×) and coded. A mouse 31-Plex cytokine/chemokine array was run by Eve Technologies (Calgary, AB, Canada) who were blinded to the identity of samples, and analytes with concentrations less than 1 pg/ml were excluded from further analysis.

### In vivo assay of the intestinal permeability

Mice had ad libitum access to food and water. Between 9 and 11 am (ZG 2–4) mice were weighted, briefly anesthetized, and gavaged with 1% body weight (volume to mass) of a cell-impermeant fluorescein-5-(and-6)-sulfonate (1 mg/ml, 478.32 Da; Life technologies corporation, Carlsbad, CA) or fluorescein isothiocyanate-dextran (FITC-Dextran, 4 kDa, Sigma Cat #46944). Blood was collected 5 h after the gavage by decapitation and left in a refrigerator (+4 C) overnight. Samples were centrifuged in a pre-chilled centrifuge (+4 C) at 3000 g for 15 min. Serum was diluted 1:2 with dd-water (50 µl of each) and fluorescence intensity was measured on a Synergy™ H1 microplate reader (BioTek Instruments, Inc., Winooski, Vermont, USA) using Gen5™ software (BioTek Instruments, Inc.) using 495/520 nm (excitation/emission) wavelengths.

### Paracellular permeability of the distal colon

Intestinal barrier function was assessed using Ussing chambers as described previously^19^. Briefly, segments of distal colon were mounted in Ussing chambers (aperture 0.3 cm^2^; EasyMount Ussing Chamber system, Physiologic Instruments, San Diego, CA, USA), equilibrated for 20 min, and a cell-impermeant fluorescein-5-(and-6)-sulfonate (478.32 Da; Life technologies corporation, Carlsbad, CA) was added to the mucosal chamber (0.05 mg ml^−1^). Samples from the serosal chamber were taken before the dye was added and every 20 min for 2 h (100 μL duplicates and buffer was replenished). Fluorescence intensity was measured on a Synergy™ H1 microplate reader using Gen5™ software using 495/520 nm (excitation/emission) wavelengths. Intestinal permeability was assessed from the slope of fluorescence values of the last four time points. Experiments were performed in a blinded fashion where the experimenter was unaware of the genotype, DSS treatment, or neutralization antibody injections.

### In vivo neutralization

One day after the completion of the DSS treatment, mice received an intraperitoneal injection of 100 µg of either a rat anti-mouse CXCL1 (R and D Systems Cat# MAB453, RRID: AB_2087696) or a rat IgG2A isotype control (R and D Systems Cat# MAB006, RRID: AB_357349). Intestinal barrier function was assessed 3 weeks after the treatments using Ussing chambers as described above.

### Flow cytometry of mouse colon lamina propria

Leukocytes from the colon lamina propria were isolated using a modification of a published protocol^58^. Briefly, colons were harvested and flushed with ice-cold Epithelial Removal Buffer (ERB) composed of HBSS without Ca^2+^ or Mg^2+^ (Cat. No. 14185, Thermo Fisher Scientific) and supplemented with 10 mM HEPES and 5 mM EDTA. After removing the remaining mesentery, colon tissues were cut into smaller pieces, placed into a conical tube, and incubated with the fresh aliquot of the ERB for 10 min at 4 °C with occasional mixing. The tissues were transferred into another fresh aliquot of the ERB and the process (incubation for 10 min at 4 °C, vortexing, and the tissue transfer) was repeated for additional 2 times. Then, preparations were rinsed twice with a regular HBSS (Cat. No. 14065, Thermo Fisher Scientific) substituted with 10 mM HEPES and digested in this buffer substituted with Liberase TM (0.13 Wünsch units/ml) and DNAse I (100 Kuntz units/ml) for 30 min at 37 °C with occasional mixing. A gentleMACS Dissociator and gentleMACS C tubes were used for cell dissociation. The enzyme reaction was stopped by an ice-cold ERB substituted with 10% fetal bovine (FBS, Denville Scientific, Inc). Cell suspensions were filtered through 100 µm and 40 µm cell strainers. Filtered cell suspensions were spun down (10 min at 350 × *g* and 4 °C), resuspended, and aliquoted in 1.5 ml centrifuge tubes containing 1 ml of PBS at RT. Cells were resuspended in 100 ul PBS containing Zombie Yellow™ Fixable Viability dye (BioLegend cat # 423103, diluted 1:500) for 15 min at RT, then washed with 1 ml of PBS and spun down at 400 × *g* for 5 min at 4 °C (following washing/centrifugation steps had the same conditions). Cells were incubated with flow cytometry buffer (FCB, filtered 1% bovine serum albumin in PBS) containing TruStain FcX™ (anti-mouse CD16/32 antibody, Biolegend cat # 101319, 1:100) for 15 min at 4 °C. Primary conjugated antibodies (Table S5) were added and incubated for 1 h at 4 °C. Cells were washed twice with FCB, resuspended in 300 µl of FCB, and analyzed on a BD LSR II [Becton Dickinson (BD) Biosciences, San Jose, CA] run by BD FACSDiva v8.0.1. Of note, a regular AmCyan bandpass filter was exchanged for 585/42. Compensation was performed using unstained cells and single-stained beads. UltraComp eBeads™ Compensation Beads (Invitrogen™ cat # 01-2222-42) and ArC™ Amine Reactive Compensation Bead Kit (Thermo Scientific cat # A10346)were used for conjugated antibodies and Zombie NIR ™ Fixable Viability dye. Each fully stained sample was run acquiring 20,000 events. Fluorescence minus one (FMO) controls were run for proper gating. Post-hoc analysis was performed using FCS Express 7 Research Edition (Win64) v7.01.0018.

### Primary cultures of mouse enteric glia

Enteric glia were isolated and cultured as described elsewhere^34^. Briefly, colons were flushed with ice-cold DMEM/Nutrient Mixture F-12 (Life Technologies) supplemented with L-glutamine and HEPES, the remaining mesentery was removed, and colons were cut in half to yield two 3–4 cm segments. The segments were individually placed on plastic rods (≈2 mm in diameter) and cotton swabs wetted with the ice-cold DMEM were used to remove the myenteric plexus and longitudinal muscle layers. Preparations were rinsed twice with HBSS substituted with HEPES (10 mM) and digested in 2.5 ml of HBSS/HEPES buffer substituted with Liberase TM (0.13 Wünsch units/ml) and DNAse I (100 Kuntz units/ml) for 30 min at 37 °C with occasional mixing. A gentleMACS Dissociator and gentleMACS C Tubes were used for cell dissociation. The enzyme reaction was stopped by an ice-cold complete medium composed of DMEM/Nutrient Mixture F-12 supplemented with L-glutamine and HEPES, penicillin and streptomycin (100 U/mL and 100 µg/mL), and 10% fetal bovine serum (FBS, Denville Scientific, Inc). Cell suspensions were filtered through a 100 µm cell strainer and plated in 24-well plates on coverslips coated with poly D-Lysine (100 µg/ml) and laminin (25 µg/ml). Cell suspensions were initially seeded in the complete medium and plates were placed in an incubator (37 °C, 95% O_2_/5% CO_2_). New media composed of DMEM/Nutrient Mixture F-12 supplemented with L-glutamine and HEPES, and substituted with antibiotics (penicillin 100 U/mL and streptomycin 100 µg/mL), Gibco™ N-2 Supplement (0.2%), Gibco™ G-5 Supplement (0.2%), and mouse NGF-β (0.05%) was exchanged every 2 days. Cells reached 50–80% confluency with 2 weeks. Experimental drugs including IL-1β (1 ng/ml), PSB 603 (1 µM), NECA (10 µM), PSB 603 and NECA, PSB 603 and IL-1β, or vehicle were added to media the day before collection for RNA isolation and analysis by quantitative PCR (qPCR) as described below.

### Isolation of RNA and RT–qPCR

TRIzol™ Reagent (Thermo Fisher Scientific, 15596026) or RLT buffer were used for RNA extraction from frozen tissue and cultured glia, respectively, in concert with RNeasy Mini Kit (Cat. No. 74104, QIAGEN) and DNase I (Cat. No. 79254, QIAGEN) following manufactures instructions. The concentration of the isolated RNA was determined on NanoDrop™ Lite Spectrophotometer (ThermoFisher Scientific). The same amount of total RNA per experiment was used for generation of cDNA utilizing High-Capacity cDNA Reverse Transcription Kit (Cat. No. 4368814, ThermoFisher Scientific) and Bio-Rad T100 Thermal Cycler running the following program: 25 °C for 10 min, 37 °C for 120 min, 85 °C for 5 min and hold at 4 °C. Diluted cDNA (1:5) was used as a template for quantitative PCR (qPCR) reactions utilizing *Power* SYBR™ Green PCR Master Mix (Cat. No. 4367659, ThermoFisher Scientific) and primer sets (Tables S6–7) purchased from Integrated DNA Technologies, Inc. The reactions were run in the Research Technology Support Facility Genomics core at MSU on a QuantStudio 7 Flex qPCR instrument with QuantStudioTM Real-Time PCR Software v1.1. All reactions following cycling conditions: 1× (50 °C for 2 min), 1× (95 °C for 10 min), 40× (95 °C for 15 s and 60 °C for 1 min), and hold at 4 °C. All reactions also had a control ROX dye and the ROX fluorescence was unchanged in all reactions. Dissociation curves were performed at the end and data with 2 or more peaks were not included in analysis performed by QuantStudio^TM^ Real-Time PCR Software v1.7.1. Obtained *C*_*t*_ values were normalized to *Cdh17* or *18* *S rRNA* for the expression of tight junction genes in whole colon tissue or immune mediator transcript abundance in cultured glia, respectively, and presented as a relative expression from the median control sample using the standard *ΔΔC*_*t*_ method.

### Statistical analysis

Data were analyzed using Prism 7 (GraphPad Software, San Diego, CA) and are shown as mean ± standard error of the mean (SEM) unless stated otherwise such as mean ± standard deviation (SD), median ± range, and median ± 95% confidence interval. The DSS-treated group was assigned by splitting the same genotype and sex littermates into two cohorts and treating one of them with DSS. The DSS-treated cohort had an extra member if the total of the same genotype and sex littermates was an odd number. Male and female data were pooled and tested for normality using D’Agostino and Pearson or Shapiro–Wilk tests. Outliers were excluded using the ROUT method (Q = 5%). Sample sizes were decided based on prior published studies. Randomization and confounders were not controlled. Data were analyzed by two-way analysis of variance (ANOVA) followed by Tukey, Bonferroni, or Sidak post hoc tests or when applicable a two-tailed Welch’s *t*-test and one-tailed one-sample *t*-test. A *P* value of less than 0.05 was considered significantly different from the null hypothesis. Statistical details of experiments such as the statistical tests used, the exact value of n and what n represents, the definition of center and dispersion/precision measures (e.g., mean ± SD) can be found in the figure legends, figures, and Results.

## Supplementary information

Movie S1

Supplementary Information
