# Supplementary material for: Enteric glial adenosine 2B receptor signaling mediates persistent epithelial barrier dysfunction following acute DSS colitis
Source: Mucosal Immunol. 2022 Jul 22;15(5):964–76. doi: 10.1038/s41385-022-00550-7 (PMC9385475; doi:10.1038/s41385-022-00550-7)
Supplement: Supplementary file 2 — Supplementary Information [file 41385_2022_550_MOESM2_ESM.pdf]

Supplementary information for:

Title: Enteric glial adenosine 2B receptor signaling mediates persistent epithelial barrier dysfunction following acute DSS colitis

Authors: Vladimir Grubišić, Vedrana Bali, David E Fried, Holger K. Eltzschig, Simon C. Robson, Michelle Mazei-Robison, and Brian D. Gulbransen

SUPPLEMENTARY MATERIALS AND METHODS

Myeloperoxidase (MPO) Activity Assay

Distal colon tissue homogenates were assayed using the Myeloperoxidase (MPO) Activity Assay Kit (Fluorometric), Abcam Cat# ab111749 (Waltham, MA, USA). Sample preparation and the assay were performed as described in the kit protocol. Fluorescence intensity was measured on an Infinite M1000 PRO microplate reader (Tecan Group Ltd, Mannedorf, Switzerland) using i-control™ microplate reader software (Tecan, version 1.6.19.2).

Transmural conductivity (G_T) of mouse distal colon preparations

The experiments were performed as previously described[1]. Briefly, full-thickness preparations of distal colon were mounted in Ussing chambers (aperture 0.3 cm2; EasyMount Ussing chamber system, Physiologic Instruments, Reno, NV, USA) equipped with Multichannel Voltage/Current Clamp (Physiologic Instruments) and operated with Acquire & Analyse software version 2.3 (Physiologic Instruments) for automated data acquisition. The tissues were first equilibrated for 30 min in warmed (37°C) and oxygenated (5% CO2, 95% O2) Krebs buffer consisting of (in mm): 121 NaCl, 5.9 KCl, 2.5 CaCl2, 1.2 MgCl2, 1.2 NaH2PO4, 25 NaHCO3 and 11 glucose. Transmural conductance (G_T) was determined by automated 5 mV pulses and recording the resulting change in the net movement of ions across the epithelium also known as short-circuit current (I_{SC}): $G_T = I_{SC} / 5$ mV.

Visceromotor Responses (VMR) to Colorectal Distention (CRD)

VMR recordings were performed as described and validated elsewhere[2]. Briefly, mice were anesthetized using isoflurane inhalation (2.5% and 1.5 % in O_2 for induction and maintenance, respectively) and a balloon-pressure sensor was inserted into the colon approximately 1 cm from the rectum. The balloon-pressure sensor consisted of a balloon (1 cm width x 2 cm length)

secured 1 cm distal from the tip of a pressure transducer catheter (SPR-524 Mikro-Tip catheter; Millar Instruments, Houston, TX, USA). Each balloon was connected to a barostat and the pressure transducer was connected to a preamplifier (model 600; Millar Instruments, Houston, TX, USA). The CRD protocol consisted of a series of three consecutive graded phasic distention periods at a constant pressure of 15, 30, 45, and 60 mm Hg with a 10 s stimulus duration and a 4-minute interstimulus interval. VMR from these trials were recorded and analyzed in a blinded fashion using LabChart 7 software (ADInstruments, Colorado Springs CO).

Social interaction (SI) test

Testing was performed as previously described[3]. Prior to testing animals were group housed. For the test, experimental animals were placed in a 42 x 42 cm arena with a mesh cylinder located at middle of one wall. Position and locomotor activity was quantified using video tracking software (TopScan, CleverSys). In the first session, animals were allowed 2.5 minutes to explore and habituate to the arena (target absent). In the subsequent 2.5min session, a novel target mouse was placed into the mesh cage and the experimental animal was allowed to freely move around the arena (target present). Time spent within an 8 cm radius of the mesh cylinder (the interaction zone) was recorded for both sessions. SI ratio was calculated as the time spent in the interaction zone with the target animal present divided by the time spent in the interaction zone with the target absent.

Table S1. Glial A_{2B}Rs distinctly mediate inflammation-induced production of key immune mediators by enteric glia and consequently broadly modulate immune signaling in the mouse colon.

RNA/protein expression of immune mediators	Glial A2BR-mediated production	
	Enteric glia*	Colon tissue**
Ccl11 / CCL11		↑
Csf3 / CSF3	↓	↑
Cxcl1 / CXCL1	↓	↑
Cxcl9 / CXCL9		↑
Cxcl10 / CXCL10	↓	↑
Il1a / IL-1a		↑
Il6 / IL-6	↑	↑
Il12b / IL-12b		↑
Il17a / IL-17a		↑

*, IL-1b-induced primary cultures of enteric glia. Of note, IL-6 has pleiotropic effects on various immune and other cells and CSF3/CXCL1 or CXCL10 are primarily reserved for granulocytes or macrophages/lymphocytes.

**, DSS-colitis induced in vivo. Since immune mediators can be produced by many different cell types such as immunocytes and epithelial/endothelial cells in the gut wall, the net production is an outcome of intercellular communication.

Table S2. Details of primary antibodies used for immunohistochemistry.

Antibody	Vendor and Catalog No.	RRID	Dilution
Biotinylated mouse anti-HuC/D	Innovative Research Cat# A21272	AB_1500232	1:200
Chicken anti-GFAP	Abcam Cat# ab4674	AB_304558	1:1000
Rabbit anti-Adenosine A2b Receptor	Millipore Cat# AB1589P	AB_2226537	1:50
Rabbit anti-Ki67	Abcam Cat# ab15580	AB_443209	1:500
Rabbit anti-cAMP	Millipore Cat# 07-1497	AB_10616218	1:100
Rabbit anti-Claudin-1	BiCell Scientific Cat# 00201	AB_2893423	1:100
Rabbit anti-S100 beta [EP1576Y]	Abcam Cat# ab52642	AB_882426	1:200
Rat anti-CD45	BD Biosciences Cat# 550539	AB_2174426	1:300
Rat anti-Occludin (N-terminus)	BiCell Scientific Cat# 00241n	AB_2893424	1:100

CD, a cluster of differentiation; GFAP, glial fibrillary acidic protein.

Table S3. Details of secondary antibodies used for immunohistochemistry.

Antibody	Vendor and Catalog No.	RRID	Dilution
Alexa 488-conjugated Donkey Anti-Chicken	Jackson ImmunoResearch Labs Cat# 703-545-155	AB_2340375	1:400
Alexa 488-conjugated Goat Anti-Chicken	Molecular Probes Cat# A-11039	AB_142924	1:400
Alexa 488-conjugated Goat anti-Rabbit	Thermo Fisher Scientific Cat# A-11034	AB_2576217	1:400
Alexa 594-conjugated Donkey Anti-Rabbit	Jackson ImmunoResearch Labs Cat# 711-585-152	AB_2340621	1:400
Alexa 594-conjugated Goat Anti-Chicken	Jackson ImmunoResearch Labs Cat# 103-585-155	AB_2337391	1:400
Alexa 594-conjugated Goat Anti-Rat	Jackson ImmunoResearch Labs Cat# 112-585-003	AB_2338372	1:400
Alexa 594-conjugated streptavidin	Jackson ImmunoResearch Labs Cat# 016-580-084	AB_2337250	1:400
Cy5-conjugated Goat Anti-Rat	Jackson ImmunoResearch Labs Cat# 112-175-143	AB_2338263	1:400

Cy, cyanine.

Table S4. Histological Disease Activity Scoring.

Feature Scored	Score
epithelial damage	0-3
immune infiltration	0-3
crypt architecture	0-3
abscess present	Present (1) or absent (0)

0 = 0-5%, 1 = 6-25%, 2 = 26-50%, 3 = 51-100% of tissue affected.

Table S5. Details of conjugated primary antibodies used in flow cytometry.

Antibody	Vendor and Catalog No.	RRID	Dilution
Alexa Fluor 488 anti-mouse CD45.2	BioLegend Cat# 109816	AB_492868	1:200
APC/Cyanine7 anti-mouse CD3	BioLegend Cat# 100222	AB_2242784	1:100
Brilliant Violet 421(TM) anti-mouse Ly-6G	BioLegend Cat# 127628	AB_2562567	1:50
PE/Cyanine7 anti-mouse IL-17A	BioLegend Cat# 506922	AB_2125010	1:100

Table S6. Details of primers used for expression of selected tight junction genes.

Gene	NCBI RefSeq		Primer sequence	size of the product (bp)
18S rRNA	NR_003278.3	sense	GGGAGGTAGTGACGAAAAATAACAAT	101
		antisense	TTGCCCTCCAATGGATCCT	
Cdh17	NM_019753.4	sense	GCTACAGATCTGGATGATCCG	200
		antisense	ATGTCCTTCACCGAGACCAC	
Cldn1	NM_016674.4	sense	GGCTTCTCTGGGATGGATCG	135
		antisense	CTTTGCGAAACGCAGGACAT	
Cldn2	NM_016675.4	sense	CCGTGTTCTGCCAGGATTCTC	146
		antisense	AGGAACCAGCGGCGAGTAG	
Cldn3	NM_009902.4	sense	CCTAGGAACTGTCCAAGCCG	134
		antisense	CCCGTTTCATGGTTTGCCTG	
Cldn4	NM_009903.2	sense	CGTAGCAACGACAAGCCCTA	197
		antisense	TGTCCCCAGCAAGCAGTTAG	
Cldn5	NM_013805.4	sense	GTTAAGGCACGGGTAGCACT	137
		antisense	TACTTCTGTGACACCGGCAC	
Cldn7	NM_016887.6	sense	GCATACTTTCTGGGGGCCA	157
		antisense	TGAAGCGACACTCTCACAGC	
Cldn8	NM_018778.3	sense	AAGGTCTACGACTCCCTGCT	152
		antisense	TTCACGTTCTCATCGTCCCC	
F11r	NM_172647.2	sense	AATTGACCTGCACCTACTCTG	105
		antisense	GAGCTGTGATCTGGCTGTTAT	
Ocln	NM_001360537.1	sense	CTGACTATGCGGAAAGAGTTGAC	105
		antisense	CCAGAGGTGTTGACTTATAGAAAGAC	
Tjp1	NM_009386.2	sense	AAGAAAAAGAATGCACAGAGTTGTT	180
		antisense	GAAATCGTGCTGATGTGCCA	

Cdh, cadherin; Cldn, claudin; F11r, F11 receptor also known as Junctional adhesion molecule A (JAM-A); Ocln, Occludin; Tjp1, tight junction protein 1 also known as zonula occludens 1 (ZO-1).

Table S7. Details of primers used for expression of selected immune mediators.

Gene	NCBI RefSeq		Primer sequence (5‘ to 3‘)	size of the product (bp)
18S rRNA	NR_003278.3	Sense	GGGAGGTAGTGACGAAAAATAACAAT	101
		antisense	TTGCCCTCCAATGGATCCT	
Ccl11	NM_011330.3	Sense	TCCATCCCAACTTCCTGCTGCT	135
		antisense	CTCTTTGCCCAACCTGGTCTTG	
Il6	NM_031168	Sense	TGTGCAATGGCAATTCTGAT	156
		antisense	GGTACTCCAGAAGACCAGAGGA	
Csf3	NM_009971	Sense	ATGGCTCAACTTTCTGCCCAG	110
		antisense	CTGACAGTGACCAGGGGAAC	
Cxcl10	NM_021274	Sense	CCAAGTGCTGCCGTCATTTTC	157
		antisense	GGCTCGCAGGGATGATTTCAA	
Cxcl1	NM_008176	Sense	CTGGGATTCACCTCAAGAACATC	117
		antisense	CAGGGTCAAGGCAAGCCTC	

Ccl, Chemokine (C-C motif) ligand; Csf, colony-stimulating factor; Cxcl, chemokine (C-X-C motif) ligand; Il, interleukin.

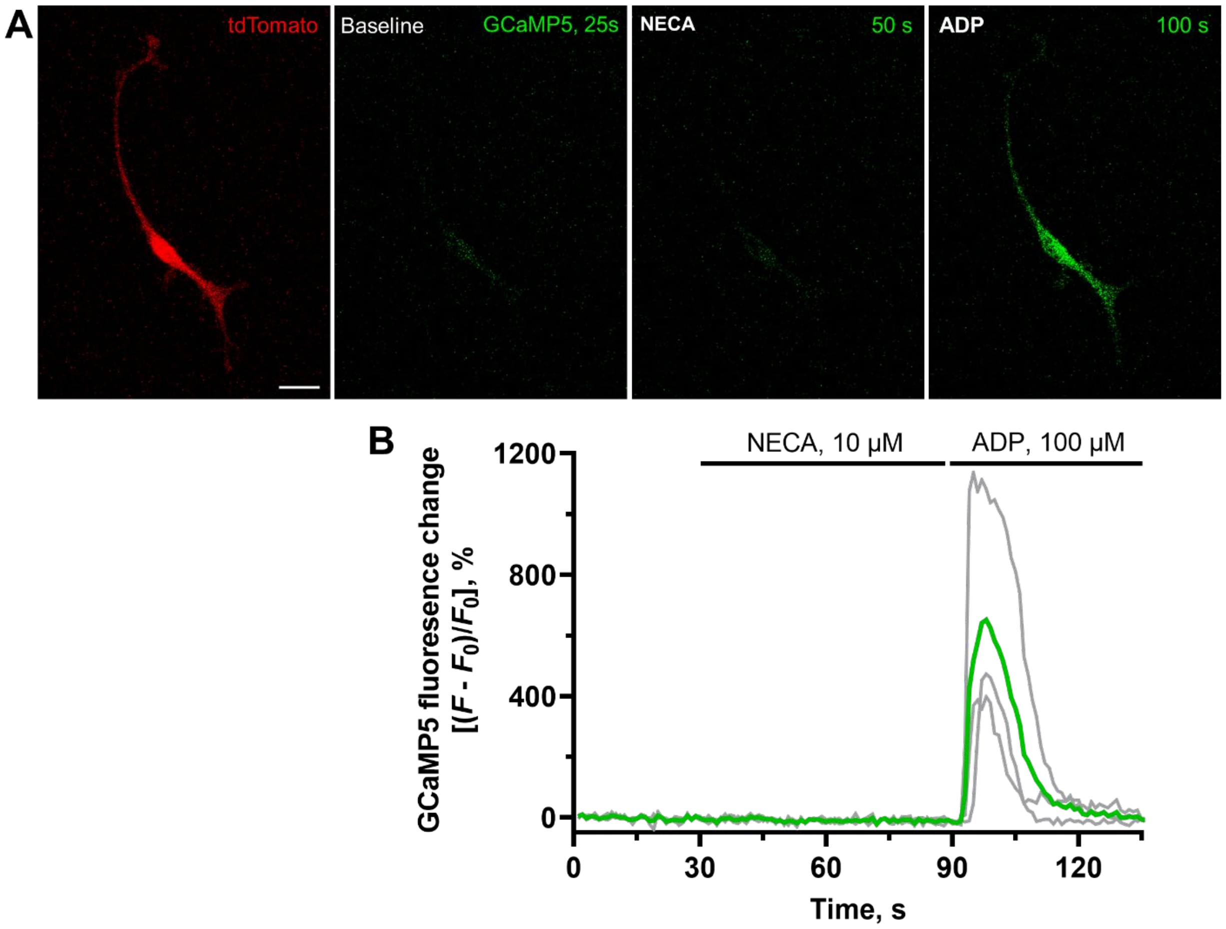

Figure S1. Calcium imaging on mouse primary enteric glia. Primary enteric glia were derived from a *Sox10*$^{CreERT2+/-}$*; GCaMP5::tdTomato*$^{f/f}$ mouse and imaged with a confocal microscope. **A)** The tdTomato fluorescence Z-projection (red) and selected frames from the GCaMP5 time-lapse imaging (green) after application of adenosine receptor agonist NECA (10 μM) and P2Y1 agonist ADP (100 μM). Numbers indicate the time of the selected frames. Scale bar = 20 μm. **B)** Summary of the GCaMP time-lapse imaging. GCaMP fluorescence (F) is analyzed as the fluorescence change from the baseline fluorescence ($F - F_0$) and normalized to the baseline fluorescence (F_0). N = 3 cells. Horizontal bars indicate the presence of NECA and ADP in the well.

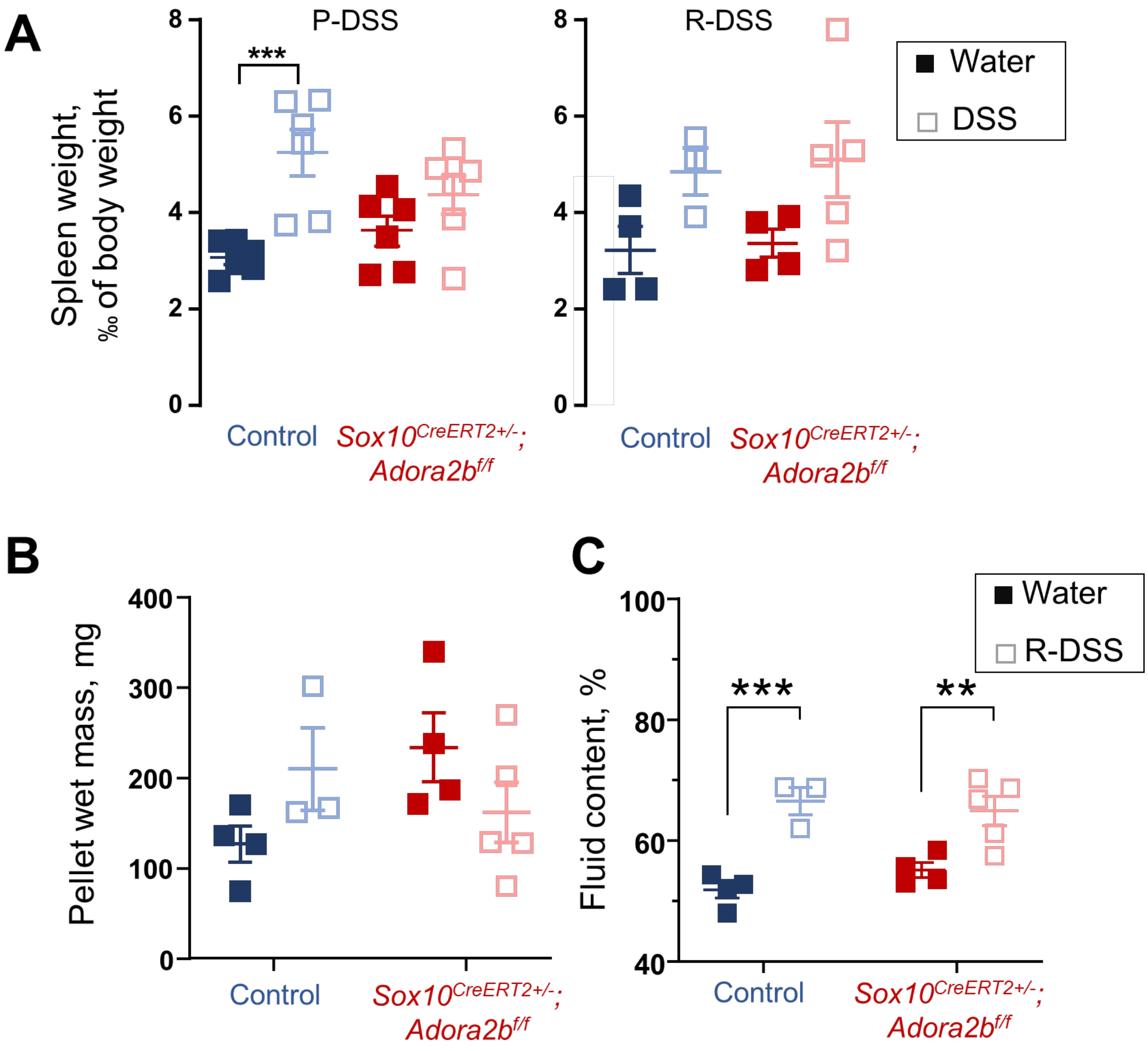

Figure S2. Spleen mass and pellet weights with fluid content . A) Normalized spleen weights at the peak (P-DSS, left) and resolution (R-DSS, right) of acute intestinal inflammation. ***, *P* = 0.0007, 2-way ANOVA, Sidak's multiple comparisons test. N = 6 mice (left) and 3-5 mice (right). **B-C)** Pellet wet mass (**B**) and fluid content (**C**) at the resolution of DSS colitis. **, *P* = 0.006; ***, *P* = 0.0007, 2-way ANOVA, Sidak's multiple comparisons test. N = 3-5 mice.

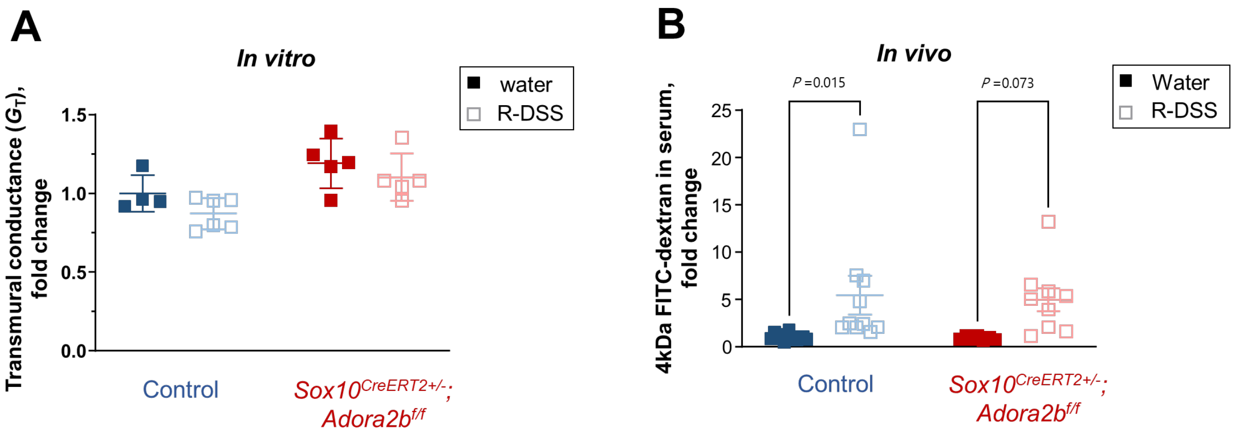

Figure S3. Additional gut permeability experiments. A) Transmural conductance (G_T) of the distal mouse colon preparations in Ussing chamber, normalized to healthy controls. 2-way ANOVA (comparisons between the Treatment, Genotype, and Interaction were $P = 0.09$, 0.03, and 0.736), Sidak's multiple comparisons between the healthy and DSS-treated animals within the same genotype were $P = 0.281$ and 0.527 for Controls and glial A_{2B}R knockouts. N = 4-6 mice. **B)** In vivo permeability to 4 kDa FITC-dextran, normalized to healthy controls. 2-way ANOVA, Sidak's multiple comparisons between the healthy and DSS-treated animals within the same genotype were $P = 0.015$ and 0.073 for Controls and glial A_{2B}R knockouts. N = 7-13 mice.

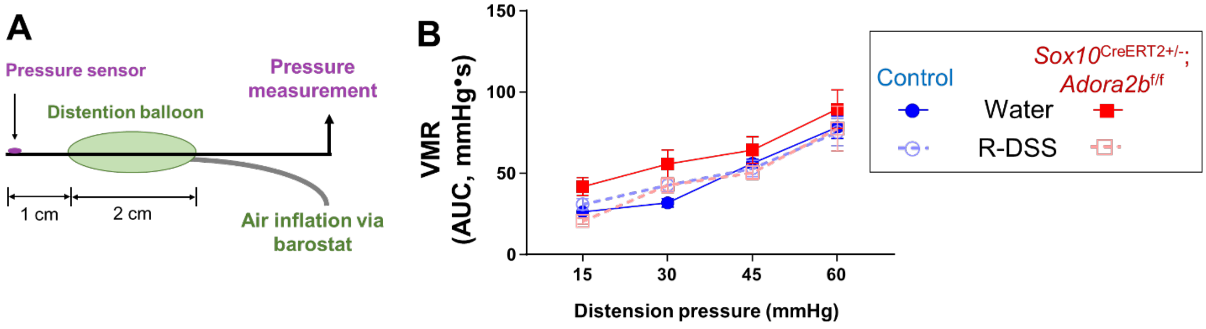

Figure S4. Mice have comparable visceral sensitivity 3 weeks after treatment with a low dose DSS. A) Model of the pressure probe and distension balloon used to record visceromotor responses (VMR) to colorectal distensions in mice. **B)** VMR in healthy and DSS-treated *Sox10*$^{CreERT2+/-}$*;Adora2b*$^{f/f}$ mice (red) and their control littermates (blue). N = 7-17 mice per group.

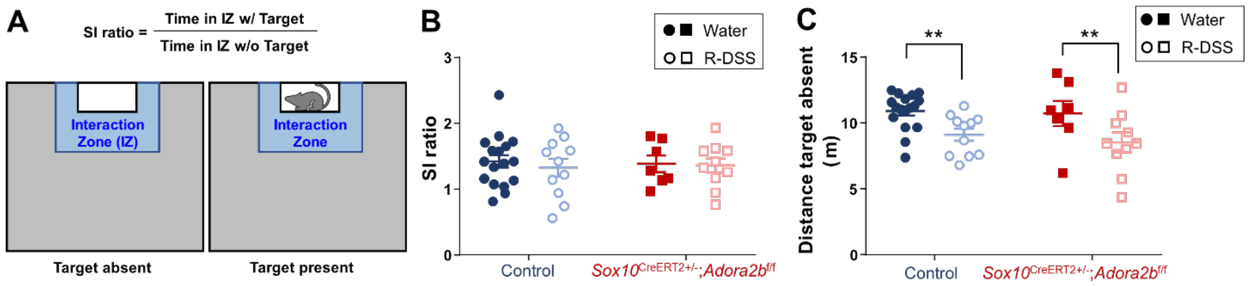

Figure S5. Mice have comparable social interaction after resolution of colitis 3 weeks after resolution of low dose DSS colitis. A) A schematic describing social interaction (SI) ratio as a measure of depressive-like behaviors in mice. An experimental mouse is first recorded in an empty arena and then with a novel "target" mouse. SI ratio is the time that mice spend in the interaction zone (IZ) with the target over the time in IZ in an empty arena. **B-C)** While SI ratios were comparable between the healthy and DSS-treated *Sox10*$^{CreERT2+/-}$*;Adora2b*$^{f/f}$ mice (red) and their control littermates (blue) **(B)**, the DSS-treated mice moved less in an empty arena **(C)**. P = 0.0013, 2-way ANOVA for the combined effect of the DSS treatment. N = 7-17 mice per group.

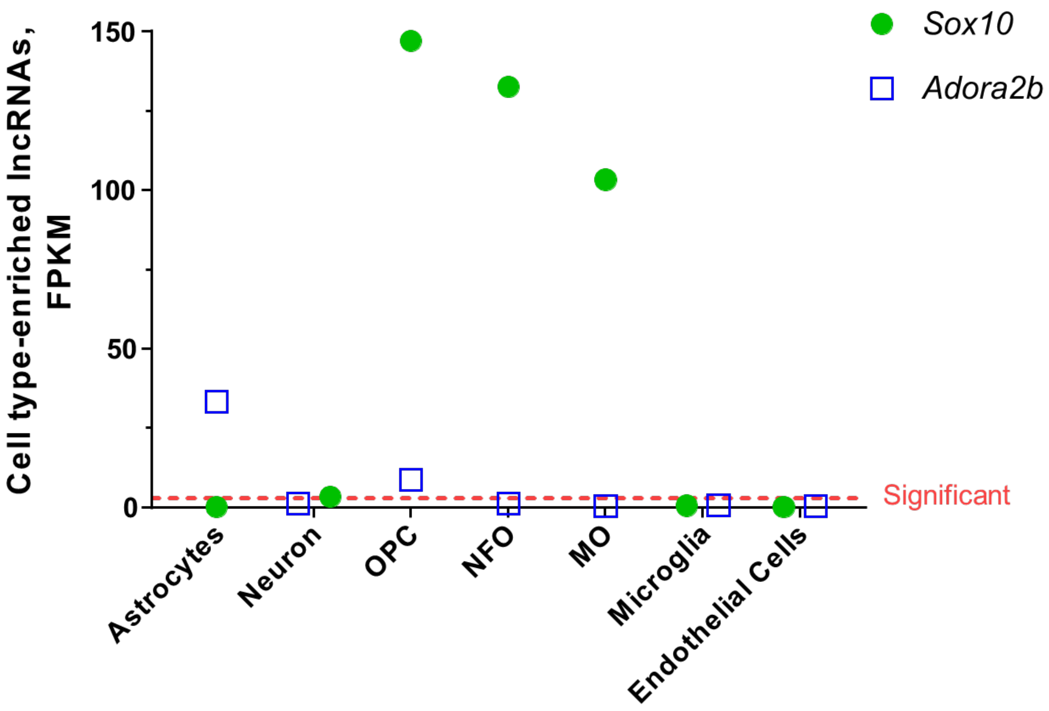

Figure S6. ***In silico*** **analysis of** ***Adora2b*** **and** ***Sox10*** **expression in the central nervous system.** Redd dotted line shows significant transcript expression levels above 3 fragments per kilobase of transcript sequence per million mapped fragments (FPKM). Therefore, cells that express both of these genes could potentially undergo tamoxifen-induced deletion of the A2BR encoding gene (*Adora2b*). In the brain, only OPC* (oligodendrocyte precursor cells) express both *Sox10* and *Adora2b* in the brain. Newly formed oligodendrocytes (NFO) and mature oligodendrocytes (MO) do not express *Adora2b* and astrocytes express *Adora2b*, but not *Sox10*. Dataset from Zhang et al 2014, *J Neurosci.* 2014 Sep 3;34(36):11929-47. doi: 10.1523/JNEUROSCI.1860-14.2014.

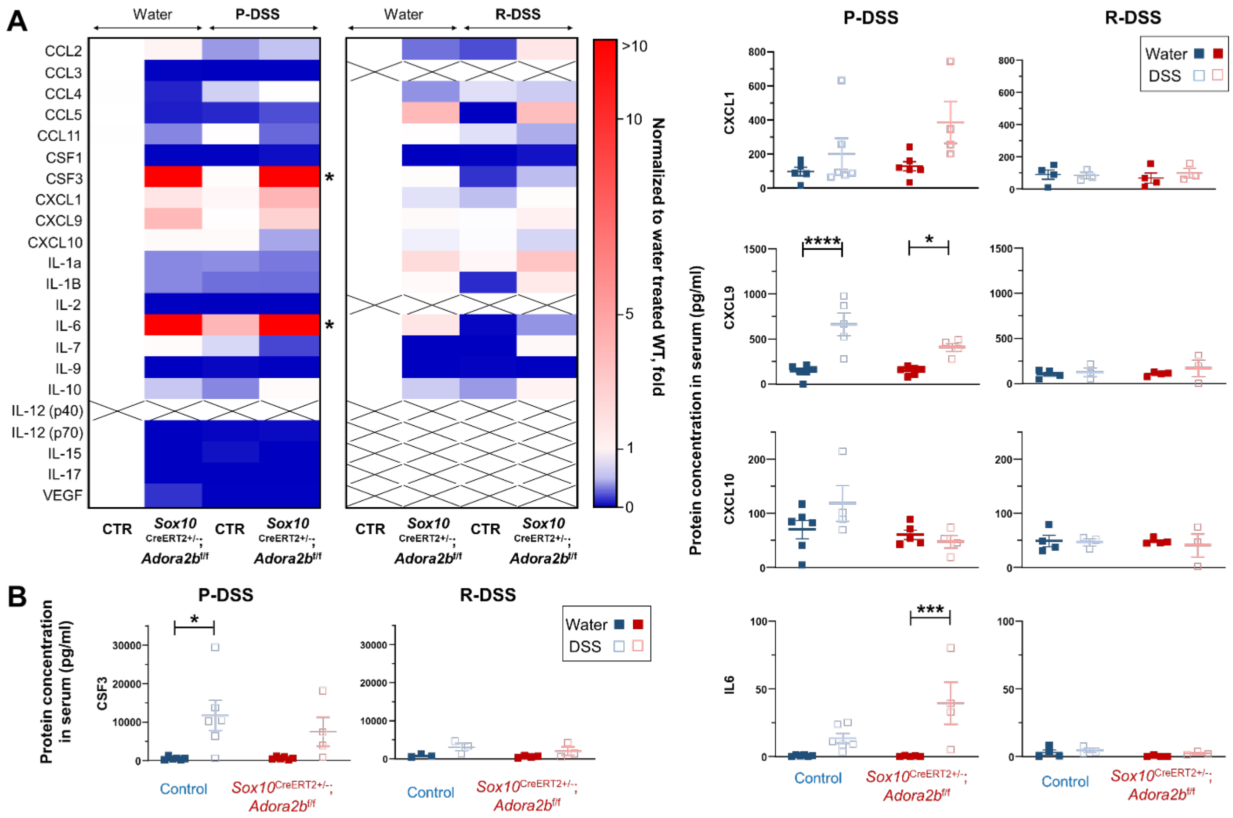

Figure S7. Immune mediators in serum are differently regulated than in the colon tissues. Multiplex assay of mouse serums after the peak (P-DSS) and resolution of acute intestinal inflammation (R-DSS). **A)** A heatmap showing changes in protein expression relative to healthy controls at P-DSS (left) and R-DSS (right). Shades of blue, white, and red depict decreased, unchanged, and increased protein abundance, respectively. Crossed-out areas indicate invalid and missing values. Asterisk and sharp symbols mark the glial A_{2B}R-dependent process as shown in Fig 5A-B. **B)** Protein concentration of CSF3, CXCL1, CXCL9, and IL-6 at P-DSS (left) and R-DSS (right). *, *P* < 0.028, ***, *P* = 0.0006, ****, *P* < 0.0001, 2-way ANOVA, Sidak's multiple comparisons test. N = 3-6 mice per group.

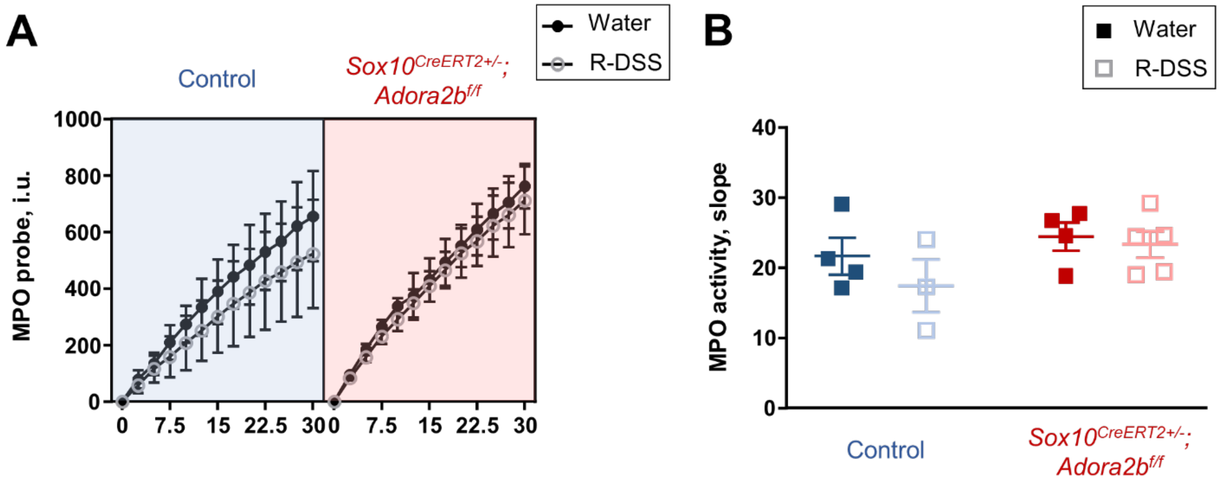

Figure S8. Myeloperoxidase activity levels in the mouse colon tissue following resolution of DSS-induced colitis. A) Myeloperoxidase (MPO) probe fluorescence readouts (expressed in intensity units, i.u.) from tissue homogenates of healthy (closed circles) and DSS-treated (open circles) Controls (left, blue) and glial $A_{2B}R$ knockouts (right, red). **B)** MPO activity was assessed by the slope of the original readouts in A. 2-way ANOVA (comparisons between the Treatment, Genotype, and Interaction were P = 0.305, 0.107, and 0.538). N = 3-5 mice.

Movie S1. Calcium dynamics in live intestinal preparations. This movie is a supplement to Figure 1F. Mouse colon longitudinal muscle myenteric plexus preparations were loaded with calcium indicator dye Fluo-4 and time-lapse imaging was performed acquiring an image every second. Drugs were perfused in the following order: BAY 60-6583 (1 μM) from 30 to 90 and then ADP (100 μM) from 180-210 s. The movie is compressed to 30 frames per second. The image size is 277 x 234 μm.

Supplementary references:

1. Grubisic V, Gulbransen BD. Enteric glial activity regulates secretomotor function in the mouse colon but does not acutely affect gut permeability. J Physiol 2017; 595(11): 3409-3424.

2. Larauche M, Gourcerol G, Million M, Adelson DW, Tache Y. Repeated psychological stress-induced alterations of visceral sensitivity and colonic motor functions in mice: influence of surgery and postoperative single housing on visceromotor responses. *Stress* 2010; **13**(4)**:** 343-354.

3. Cooper SE, Kechner M, Caraballo-Perez D, Kaska S, Robison AJ, Mazei-Robison MS. Comparison of chronic physical and emotional social defeat stress effects on mesocorticolimbic circuit activation and voluntary consumption of morphine. *Sci Rep* 2017; **7**(1)**:** 8445.
